# Competing interests during the key *N*-glycosylation of 6-chloro-7-deaza-7-iodopurine for the synthesis of 7-deaza-2′-methyladenosine using Vorbrüggen conditions

**DOI:** 10.3389/fchem.2023.1163486

**Published:** 2023-03-23

**Authors:** Fabrício Fredo Naciuk, Andrey Fabricio Ziem Nascimento, Rebeca Paiva Froes Rocha, Joane Kathelen Rustiguel, Lais Durço Coimbra, Rafael Elias Marques, Marjorie Bruder

**Affiliations:** ^1^ Brazilian Biosciences National Laboratory, Brazilian Center for Research in Energy and Materials, Campinas, São Paulo, Brazil; ^2^ Brazilian Synchrotron Light Laboratory, Brazilian Center for Research in Energy and Materials, Campinas, São Paulo, Brazil

**Keywords:** 7DMA, Vorbrüggen, *N*-glycosylation, TMSOTf, *N*-silyl ketene imine formation, emerging virus

## Abstract

A short 3-step synthesis of the antiviral agent 7DMA is described herein. The nature of a major by-product formed during the key *N*-glycosylation of 6-chloro-7-deaza-7-iodopurine with perbenzoylated 2-methyl-ribose under Vorbrüggen conditions was also investigated. Spectroscopic analyses support that the solvent itself is converted into a nucleophilic species competing with the nucleobase and further reacting with the activated riboside in an unanticipated fashion. These findings call for a revision of reaction conditions when working with weakly reactive nucleobases in the presence of Lewis acids. 7DMA thus obtained was evaluated for its efficacy against an emerging flavivirus *in vitro*.

## 1 Introduction

**FIGURE 1:**
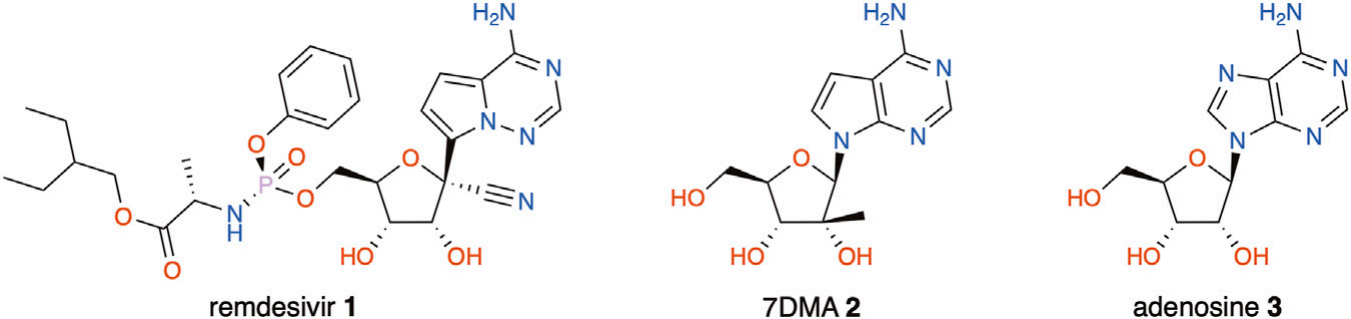
Antiviral agents remdesivir (**1**), 7DMA (**2**), and the nucleobase adenosine (**3**).

Nucleoside analogues make up a valuable chemical class in the fields of both anticancer and antiviral chemotherapy (Jordheim et al., 2013). In the latter, they may target RNA-dependent RNA polymerases (RdRp), thereby inhibiting viral replication (Carroll et al., 2003; Benzaria et al., 2007; Boehr et al., 2019). This strategy remains effective for emerging RNA viruses, such as Zika (ZIKV), West Nile (WNV), and Ebola (EBOV); especially with the recent coronavirus (SARS-CoV-2) outbreak, adenosine analogue remdesivir (**1**) has made a compelling case (Eyer et al., 2016, 2019; Tchesnokov et al., 2019; Gordon et al., 2020; Yin et al., 2020). Another modified nucleoside that is frequently used as a tool in flavivirus research is 7-deaza-2′-*C*-methyladenosine (7DMA, **2**), a close analogue to adenosine (**3**) (Figure 1). 7DMA was originally developed by Merck (under the name MK-608) to target the RdRp (NSB5) of the hepatitis C virus (HCV) and had shown promising results before failing in clinical trials (Carroll et al., 2009; Arnold et al., 2012). Still, it is frequently used to study other *Flaviviridae* viruses such as the dengue virus (DENV) (Schul et al., 2007), tick-borne encephalitis virus (TBEV) (Eyer et al., 2015), and ZIKV (Zmurko et al., 2016; Eyer et al., 2019; Jacobs et al., 2019).

**SCHEME 1:**
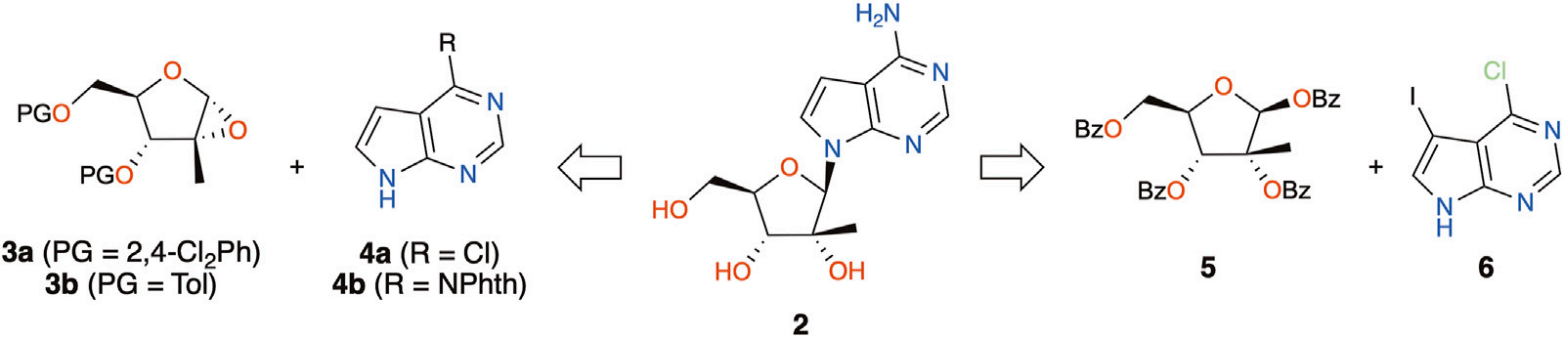
Previous and proposed synthetic routes toward 7DMA (**2**).

In view of using 7DMA to study viral metabolism at our research institute, and given the high cost of commercial sources, our chemistry group set out to produce the compound in house. Only two research articles disclosed in detail the synthesis of 7DMA. Eldrup and colleagues used an *in situ* generated α-epoxide intermediate (Scheme 1, **3a**) to react with the sodium salt of 7-deazaadenine (**4a**) (Eldrup et al., 2004), whereas Bio and co-workers sought to generate the α-epoxide (**3b**) prior to undergoing aminolysis by the sodium salt of *N*6-protected 7-deazaadenine (**4b**) (Bio et al., 2004). About a dozen patent applications filed between 2002 and 2010 report the synthesis based on these approaches; however, the preparation of the key epoxide intermediates can require 5 to 10 steps. Looking for a shorter process to produce compound **2**, we envisioned the traditional Vorbrüggen protocol for the key *N*-glycosylation reaction (Vorbrüggen and Ruh-Pohlenz, 1999; Beutner et al., 2019). Although Bio and co-workers have described the lack of reactivity of 6-chloro-7-deazapurine (**4a**) under these conditions (Bio et al., 2004), the Hocek group succeeded in coupling perbenzoylated 2-methyl-ribose (**5**) with iodinated 6-chloro-7-deazapurine (**6**) (Scheme 1) in the presence of TMSOTf and DBU in 48% yield (Nauš et al., 2012). In 2020, Cho also reported the same coupling in 73% yield using TMSOTf and BSA as activating agents (Cho et al., 2020). While in these studies, the iodine group was used as a handle for further cross-coupling reactions, the latter halogen could also be removed by simple catalytic hydrogenation after a one-pot chlorine–nitrogen exchange and removal of the benzoyl groups in aqueous ammonia, thus yielding 7DMA (**2**). As such, we describe a short synthesis of 7DMA using the Vorbrüggen protocol and an unexpected side-reaction that occurred during this key step. The *in vitro* activity of 7DMA against the emerging mosquito-borne Usutu virus (USUV) is also reported.

## 2 Materials and methods

### 2.1 Synthetic procedure for compound 10

Please refer to the Supplementary Material for general synthetic and analytical procedures for the known compounds **7a**, **8**, **8a**, **5**, **6**, **9**, **13**, and **2**.

(6-(Benzoyloxy)-2-(cyanomethyl)-6a-methyl-2-phenyltetrahydrofuro[2,3-*d*][1,3]dioxol-5-yl)methyl benzoate **(10)**.

To a mixture of **5** (2.65 g, 4.57 mmol), **6** (1.53 g, 5.49 mmol, 1.2 equiv.) and DBU (2.10 mL, 13.72 mmol, 3.0 equiv.) in acetonitrile (46 mL) was added TMSOTf (3.30 mL, 18.30 mmol, 4.0 equiv.) dropwise at 0°C and the mixture was then stirred at 70°C for 24 h. After cooling, the mixture was diluted with ethyl acetate (45 mL) and saturated aq. NaHCO_3_ (45 mL) was added. The aqueous layer was further extracted with ethyl acetate (4 mL × 45 mL). The combined organic layers were washed with water (45 mL) and brine (45 mL), dried over MgSO_4_, and concentrated. The crude product was purified once by chromatography on silica gel (CHCl_3_/MeOH, 98:2) and once by reverse-phase chromatography (MeCN/H_2_O, 80:20) to yield the expected product (**9**) in only 20% (0.69 g, 0.93 mmol), and the by-product (title compound) (1.22 g, 2.45 mmol, 54%) in the more polar fractions. An analytical sample of **10** for the X-ray diffraction experiment was obtained by crystallization from hexane to yield colorless crystals. HRMS calcd for C_29_H_26_NO_7_^+^ [M + H]^+^: 500.1704, found: 500.1708; ^1^H NMR (500 MHz, CDCl_3_) *δ* 7.97–7.85 (m, 4H), 7.65–7.57 (m, 3H), 7.56–7.49 (m, 1H), 7.48–7.42 (m, 2H), 7.41–7.31 (m, 5H), 5.88 (s, 1H), 5.12 (d, *J* = 9.0 Hz, 1H), 4.37 (dd, *J* = 12.1, 3.5 Hz, 1H), 4.17 (dd, *J* = 12.3, 5.0 Hz, 1H), 3.65–3.59 (m, 1H), 2.92 (dd, *J* = 21.4, 12,0 Hz, 2H), and 1.88 (s, 3H). ^13^C NMR (125 MHz, CDCl_3_) δ 165.8, 165.5, 140.6, 133.6, 133.1, 130.0, 129.6, 129.5, 129.3, 128.9, 128.5, 128.4, 128.3, 125.4, 115.9, 111.6, 110.3, 88.7, 78.1, 76.0, 63.1, 33.8, and 22.3.

### 2.2 Single-crystal X-ray studies of compound 10

The crystallographic data for compound **10** were collected at 100 K on the MANACA beamline at Sirius (Brazilian Synchrotron Light Laboratory) with a PILATUS2M detector using monochromatic X-ray (0.67937 Å). The data were recorded in the rotation mode using the ω scan technique with 2θmax = 57.2° using data collection software MXCuBE (Gabadinho et al., 2010; Oscarsson et al., 2019). The MANACA is a macromolecular crystallography beamline, and to avoid the lack of completeness at high angles, we collected data from eight crystals of compound **10** mounted in random orientations. The data reduction and merging were performed using the XDS (Kabsch, 2010). The data were corrected for absorption effects using the empirical method implemented in XDS (Kabsch, 2010). The structure was solved by direct methods (intrinsic phasing) using SHELXT (Sheldrick, 2015b). The refinement was carried out by the full-matrix least-squares method with anisotropic displacement parameters for all non-hydrogen atoms based on *F* ^*2*^ using SHELXL (Sheldrick, 2015a) through the OLEX2 interface (Dolomanov et al., 2009). The hydrogens were positioned geometrically in their idealized positions (Sheldrick, 2015a). The following reflections were omitted from refinement due to bad crystal data from one dataset: −10 10 1, 8 12 0, 1–14 5, and 6 0 12. The general-purpose crystallographic tool PLATON (Spek, 2003) was used for structure validation. MERCURY was used for molecular graphics representations. Details of the data collection and refinement and additional ORTEP style view of the X-ray crystal structure and packing diagrams of compound **10** are given in the Supplementary Material.

### 2.3 Antiviral activity assessment

#### 2.3.1 Virus and cell culture

USUV 1477 (GenBank: KJ438705.1) was provided by Prof. Dr. Jonas Schmidt-Chanasit of the Bernhard Nocht Institute of Tropical Medicine, Germany. Low-passage-number viral stocks were generated in Vero CCL81 cells, titrated and stocked at −80°C. Vero (ATCC CCL-81) and SH-SY5Y cells (CRL-2266^™^) were obtained from ATCC. Vero CCL81 cells were cultured in DMEM supplemented with 10% fetal bovine sera (FBS) and antibiotics, while SH-SY5Y cells were cultured in the same media with 50% F-15. Cell cultures were kept at 37°C, 5% CO_2_.

#### 2.3.2 *In vitro* testing of 7DMA (**2**) and i7DMA (**13**)

Cells were infected with USUV at a multiplicity of infection (MOI) of 0.1 and then treated with vehicle (0.001% DMSO) or test compounds. In the dose-dependent assays, concentrations ranging from 100 to 3.1 μM of 7DMA were used. For evaluation of the antiviral effect of i7DMA, non-infected and infected cells (see above) were treated with vehicle, 7DMA or i7DMA at 10 μM. Cell culture supernatant was collected at 48 h post-infection in dose-dependent assays of 7DMA, and 24 h post-infection in antiviral effect and cell viability assay for i7DMA. Viral loads were assessed using plaque-forming assays (Rocha et al., 2021). Cell culture viability was assessed using MTT assays [3-(4,5-dimethylthiazol-2-yl)-2,5-diphenyltetrazolium bromide] according to recommendations from the manufacturer (Merck).

## 3 Results

**SCHEME 2:**
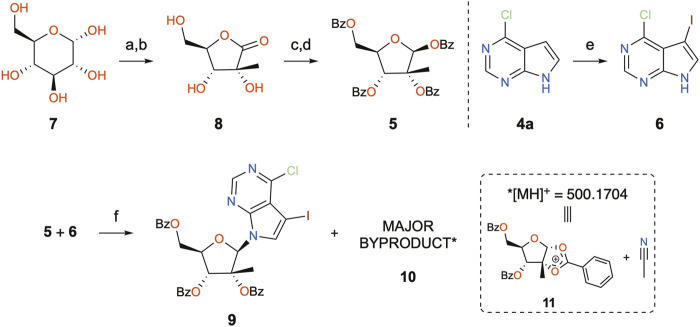
Synthetic steps toward the key Vorbrüggen *N*-glycosylation reaction. Reagents and conditions: (a) BnNHMe, EtOH, AcOH, reflux, 4 h, 52%–66% (**7a**); (b) MeOH, THF, CaCl_2_/MeONa, 40°C, 19 h, 44%–70% (**8**); (c) BzCl, Et_3_N, CH_2_Cl_2_, rt, 18 h, 58%–89% **(8a**); (d) *i*. (LiAl(O*t*Bu)_3_H), THF, rt, 3 h; *ii*. BzCl, Et_3_N, CH_2_Cl_2_, rt, 19 h, 38%–78% (**5**); (e) NIS, DMF, rt, 20 h, 81%–95% (**6**); (f) DBU, TMSOTf, MeCN, 70°C, 28 h, 20% (**9**), 54% (**10**) or DBU, TMSOTf, DCE, 70°C, 24 h, 58% (**9**).

Starting with the preparation of perbenzoylated 2-methyl-ribose (**5**) in house, D-(+)-glucose (**7**) was subjected to the Amadori reaction (Hotchkiss et al., 2006) and further treated with calcium chloride (Steinhardt and Eastgate, 2013) to give 2-methyl-D-ribonic-γ-lactone **8** (Scheme 2). Perbenzoylation of the latter prior to reduction of the lactone using lithium tri-*t*-butoxyaluminium hydride and benzoylation of the resulting crude hemiacetal furnished the desired peracylated 2-methyl-ribose **5** (Zhou et al., 2016). This 4-step sequence could be performed on a multigram scale without the use of chromatographic purification. The planned coupling partner **6** was prepared by iodination of 6-chloro-7-deazapurine **4a** (Song et al., 2011). With the key *N*-glycosylation reaction partners **5** and **6** in hand, Hocek’s protocol was followed, giving the desired product (**9**) in 48% yield (Nauš et al., 2012). On repeating the reaction, however, we only achieved low yields, around 20%. The addition of molecular sieves did not greatly improve the outcome (30% yield). Intrigued by these results, the reaction mixture was analyzed in detail. Chromatographic analyses of the reaction mixture revealed the presence of a major by-product with an ion mass ([MH]^+^) of 500.1708. Being devoid of isotopic peaks that would arise from chlorine present in **6**, potential deazapurine derivatives were dismissed. Furthermore, considering that protonated species are recorded ([MH]^+^, and the ammonium ion [MNH_4_]^+^), the corresponding molecular mass would be equal to 499, which would imply an odd number of nitrogen atoms present in the structure of the by-product (Pellegrin, 1983). As a consequence, accounting for all the reagents present in the reaction mixture that possess an odd number of nitrogen, and having excluded deazapurine derivatives, the only remaining candidate would be the solvent, acetonitrile. In consideration of the reaction intermediates generated under the reaction conditions, the activated riboside oxonium (**11**) would be the most likely reactive intermediate, and the combined masses of **11** and acetonitrile indeed had the theoretical protonated ion mass of 500.1704, the experimental data (m/z 500.1708) being within an excellent error margin (<1 ppm).

**FIGURE 2:**
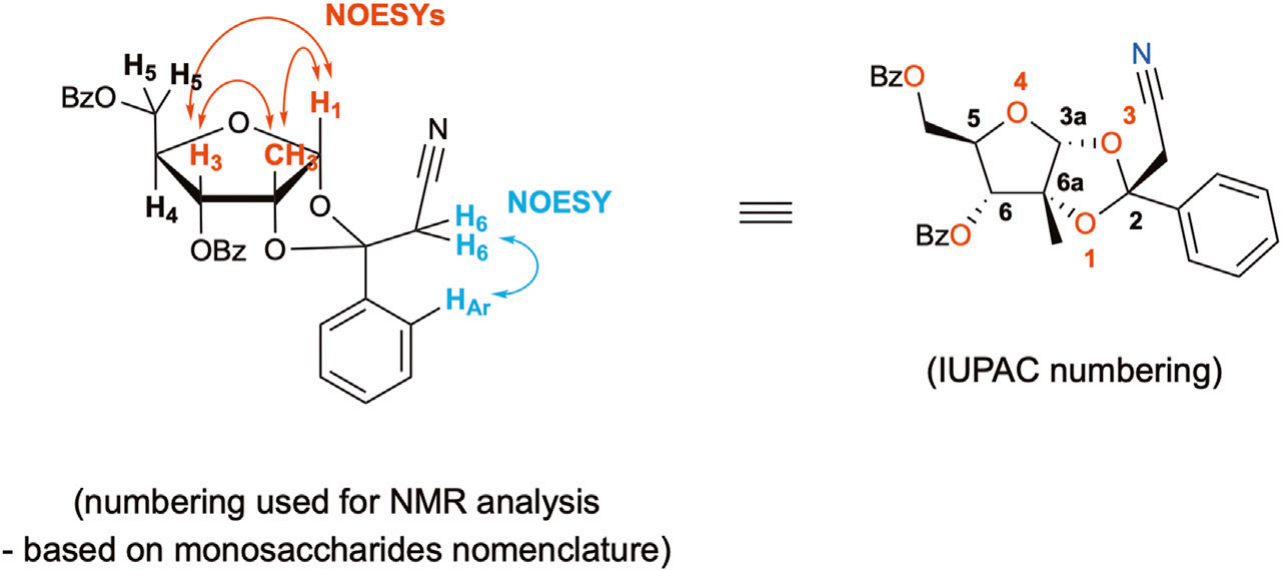
Proposed structure of by-product **10** and main NOESY correlations.

**FIGURE 3:**
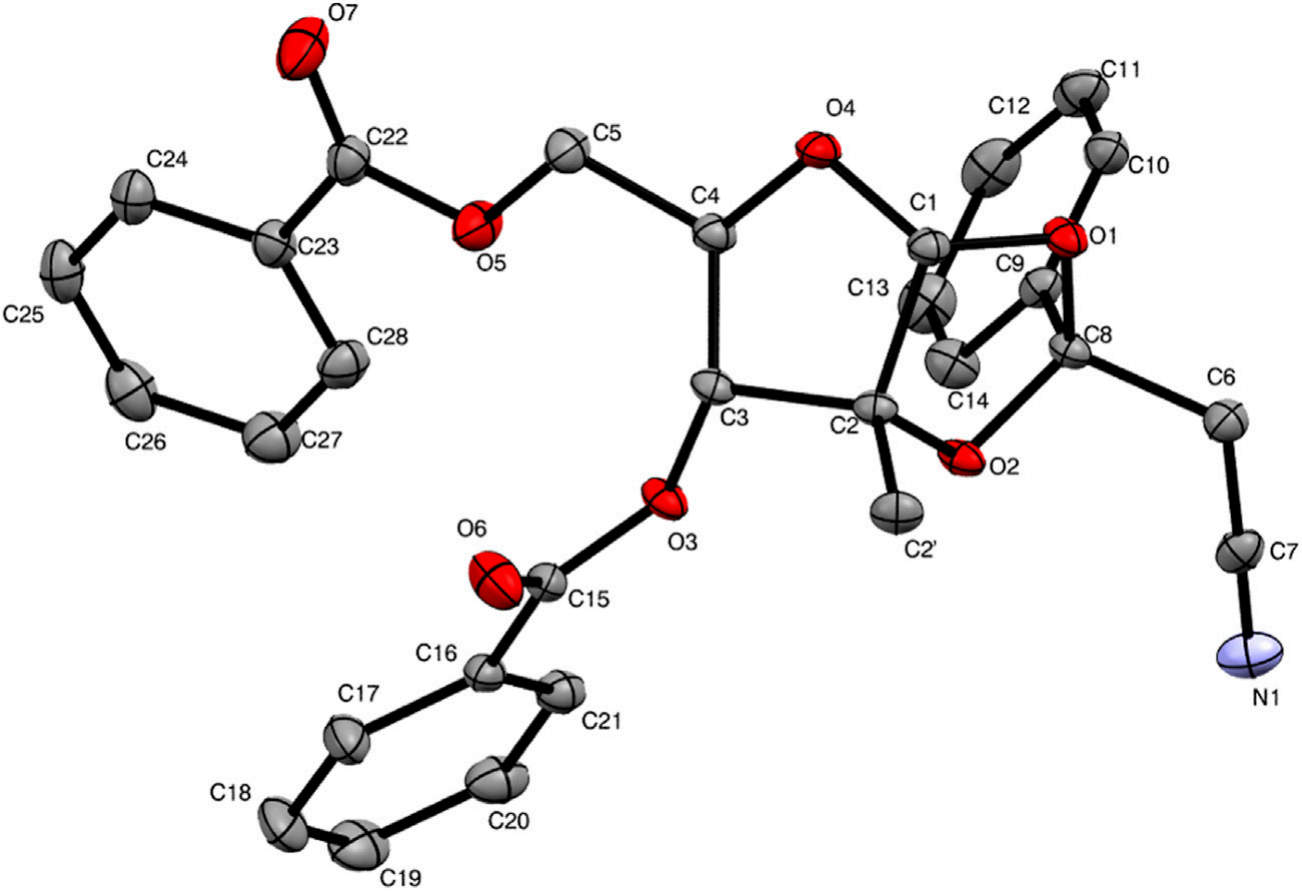
ORTEP view of compound **10**.

The by-product was further isolated in order to perform a full characterization and try to establish the causes for its occurrence. Chromatographic purification was successfully achieved, yielding a pure product with the expected mass, as examined by UPLC-HRMS analysis. The compound was thus subjected to a series of NMR experiments (please see Supplementary Material for full details), which revealed that acetonitrile was indeed connected to the 1,2-*O*-ethylidene moiety of 3,5-dibenzoyl-2-methylribose through its α-carbon. The structure of by-product **10** shown in Figure 2 has the name ((3a*R*,5*R*,6*R*,6a*R*)-6-(benzoyloxy)-2-(cyanomethyl)-6a-methyl-2-phenyltetrahydro-furo[2,3-*d*][1,3]dioxol-5-yl)methyl benzoate. Crystals of **10** suitable for X-ray crystallography diffraction experiments were also produced, which unequivocally confirmed the proposed structure, also highlighting the single stereochemistry at the ketal position, defined as *R* (Figure 3).

**SCHEME 3:**
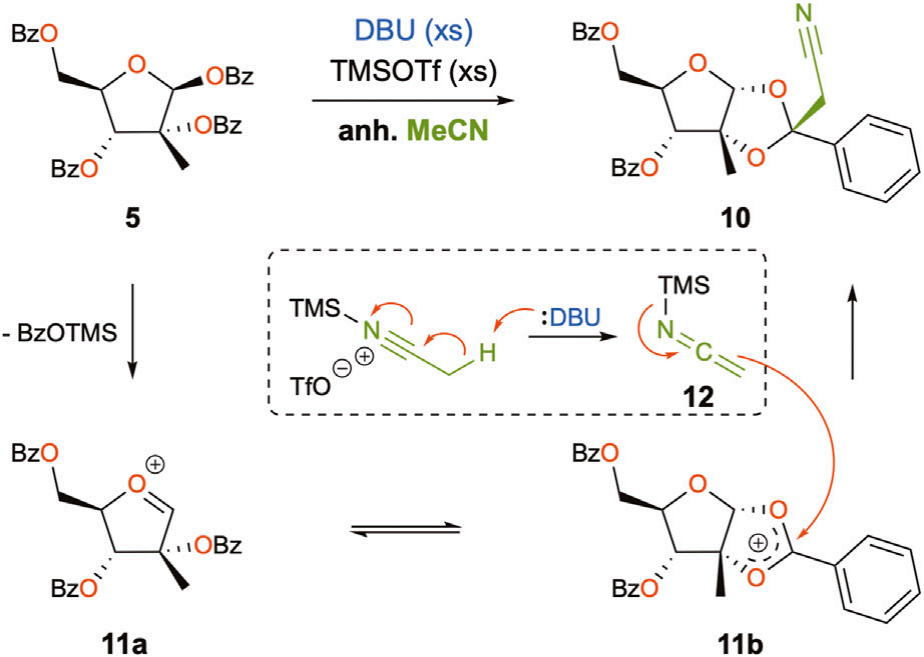
Mechanistic proposal for the formation of by-product **10**.

From a mechanistic point of view, a notable disconnection would be at the ketal moiety since the cyclic oxonium intermediate **11b** (Scheme 3) would be a plausible electrophile. In fact, although it is the first time that the alkylation of the non-anomeric position of the acyl oxonium is reported for *C* ^2^-methylated pentoses, this phenomenon has already been described during the reaction of silyl enol ethers with peracylated-D-ribofuranose catalyzed by stannic chloride to produce *C*-nucleosides (Yokoyama et al., 1982; Berber et al., 2001). Since then, a few other studies have reported this generally unexpected reactivity in *C* ^2^-acylated sugars for other *C*-nucleophiles, such as allenyltributylstannane (Chan et al., 2005), indium acetylenides (Lubin-Germain et al., 2008), trimethylaluminum (More and Campbell, 2009), cyanotrimethylsilane (Popsavin et al., 2012), or zinc acetylenides (Lemaire et al., 2014), suggesting that the small size of the nucleophile is the most decisive factor for the observed regioselectivity. The formation of the corresponding orthoesters is well known in the field of *O*-glycosylation, the latter serving as intermediates in the synthesis of oligosaccharides (Kong, 2007). Regarding the reactivity of acetonitrile under our reaction conditions, three reports describe cyanomethylation reactions with acetonitrile in the presence of a silylated Lewis acid (Iwasaki and Kume, 1987; Watanabe et al., 2017; Yoshimura et al., 2017), but it is only Yoshimura who proposes that the reactivity of alkylnitriles is mediated *via* activation by trialkylsilyl triflates, when in the presence of mild organic bases, thus generating transient *N*-silyl ketene imine nucleophiles (Yoshimura et al., 2017). As such, a mechanism for the formation of by-product **10** could be proposed, as an excess of TMSOTf and DBU was present in our reaction mixture, thus being able to generate nucleophilic acetonitrile (*via* the *N*-silyl ketene imine **12**) which would, in turn, attack intermediate **11b** at the non-anomeric position (Scheme 3). Moreover, the stereoselective attack of the least hindered β-face of **11b** could be justified. Although for most of the aforementioned cases, there was only one nucleophile in play, in our case, a competition between compounds **6** and **12** must take place, and one could assume that the lack of reactivity of nucleobase **6** favors attack by **12**. The modified Vorbrüggen protocol involving the addition of DBU was first mentioned in 1994 (Kristinsson et al., 1994), claiming the advantages of neutralization of the reaction media, in addition to promoting better solubilization of the silylated nucleobase and the dismissal of anhydrous conditions. Since then, this protocol has been described for the *N*-glycosylation of other purines (Franchetti et al., 1998; Rosenberg et al., 2003; Gunic et al., 2007).

A notable alternative would thus either be substituting acetonitrile, for example, with 1,2-dichloroethane (Smith et al., 2004), or performing the reaction without DBU. As mentioned earlier, Cho has recently reported the same coupling using BSA and TMSOTf (Cho et al., 2020) which has also been used for *N*-glycosylation reactions with other 7-deazapurine analogs, as reported by Seela and Ming (2007) and Ingale et al. ( 2018) and the Hocek group (Nauš et al., 2015; Perlíková et al., 2021) for non-*C* ^2^-branched peracylated pentoses. In our hands, however, the same side-reaction occurred on repeating Cho’s protocol. Therefore, the different reaction conditions mentioned previously were repeated and the reaction mixtures monitored by UPLC-HRMS at several time points (please see Supplementary Material for further details). Our original condition (TMSOTf/DBU/MeCN) for coupling partners **5** and **6** showed the appearance of both the desired product (**9**) and by-product (**10**) after 6 h in a 7:3 ratio which persisted overnight. On repeating Cho’s protocol with BSA/TMSOTf, almost complete conversion of the riboside was observed after 1 h; however, the reaction mixture showed the by-product (**10**) as major product, and desired product (**9**) in a 85:15 ratio, evolving to 89:11 after 6 h (Cho reported the reaction duration of 8–9 h with 73% yield) (Cho et al., 2020). This suggests that the sole presence of a Lewis acid is sufficient to promote the formation of by-product **10**. By running the DBU/TMSOTf protocol in 1,2-dichloroethane instead of acetonitrile, the desired nucleoside **9** was observed with 82% conversion after 6 h and 92% after 25 h and, as expected, with no traces of by-product **10**. This protocol was subsequently used to perform the key coupling reaction and gave compound **9** in up to 58% (mean value of 48% over four experiments), after two sequential chromatographic purifications on normal and reverse phases, respectively. Finally, the reaction of riboside **5** with 6-chloropurine using TMSOTf/DBU in acetonitrile (Bio et al., 2004) was also analyzed and showed 99% conversion to the expected product after 30 min, with no traces of by-product **10**. This observation supports that it is the lack of reactivity of nucleobase **6** that enables the generated *N*-silyl ketene imine (**12**) to attack the cyclic oxonium intermediate **11b**.

**SCHEME 4:**
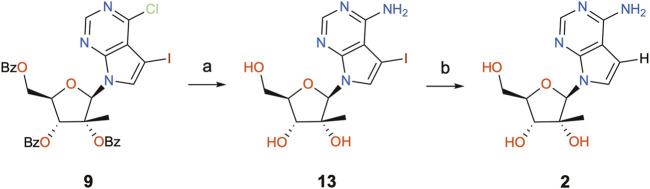
Completion of the synthesis of 7DMA. Reagents and conditions: (a) aq. NH_3_, dioxane, 120°C (sealed tube), 21°h, 42–98%; (b) H-Cube^®^ Pro reactor, 30 mm Pd/C 10% cartridge, Et_3_N, DMF/MeOH (50 mM), “full H_2_ mode”, 1 atm, 40°C, 1 mL/min flow, 77%.

In order to complete the synthesis of 7DMA (**2**), a concomitant aminolysis of the benzoyl esters and substitution of the 6-Cl atom with aqueous ammonia (Nauš et al., 2012) was performed to yield precursor **13**, followed by deiodination through catalytic hydrogenation (Scheme 4) (Nauš et al., 2015). This last step was accomplished in a flow hydrogenation reactor equipped with a Pd/C 10% cartridge under atmospheric pressure and gentle heat (40°C), whereby 1.4 g of **13** could be processed in just over 1 h, providing 750 mg of the final product (7DMA) in 77% yield after chromatographic purifications.

**FIGURE 4:**
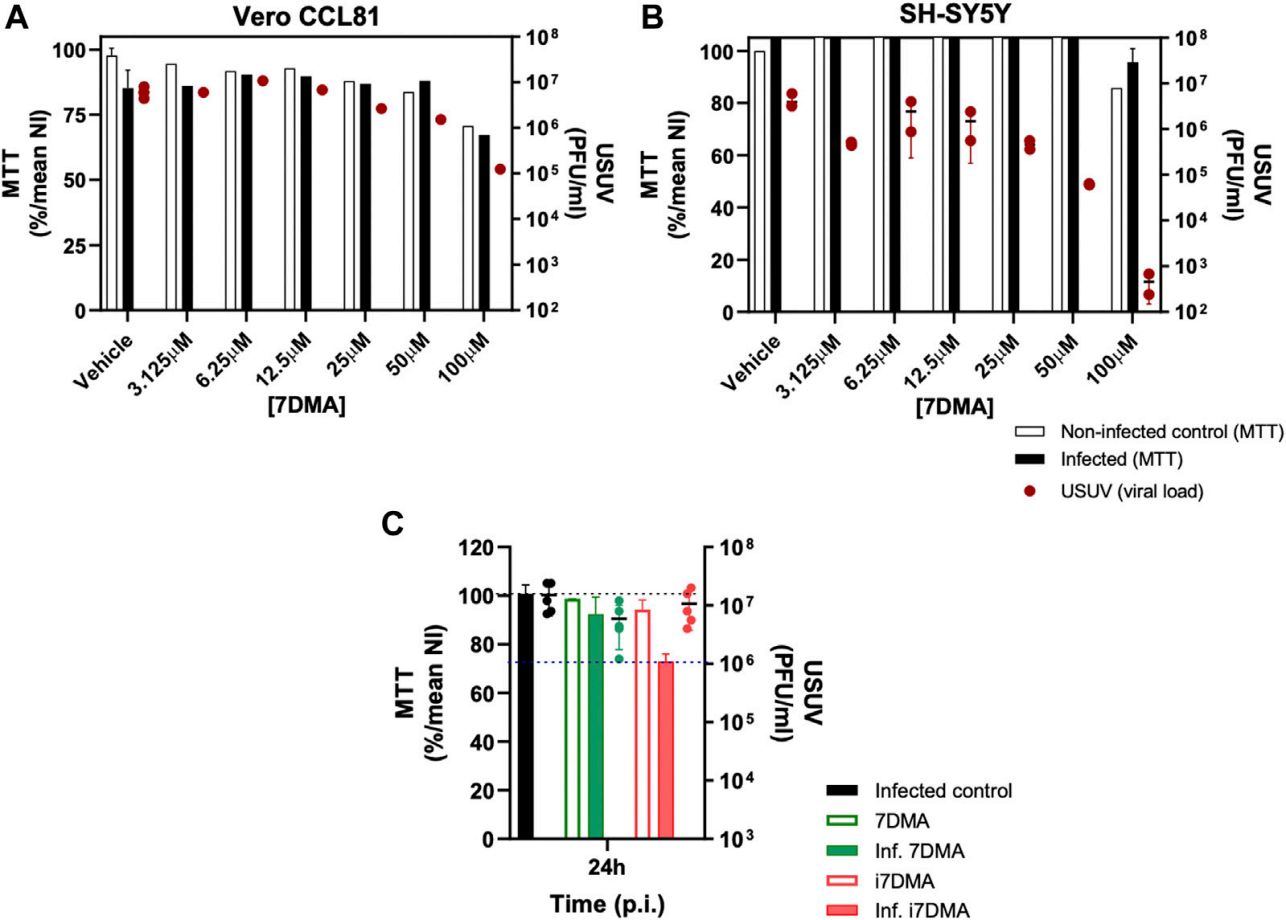
Antiviral effect of 7DMA (**2**) and i7DMA (**13**) against USUV and cell viability assessments. **(A–B)** Dose-dependent inhibition of USUV replication by 7DMA. Vero CCL81 or SH-SY5Y cells were infected and treated with vehicle or 7DMA in concentrations ranging from 3.1 to 100 µM. Cell culture supernatant was collected at 48 h post-infection for the assessment of infectious viral load. Cell viability was assessed using the MTT assay. The left Y axis represent the percentage of viable cells relative to non-infected vehicle-treated controls (NI, 100%) (white bars) and infected and treated cells (black bars). The right Y axis represent viral load results expressed in plaque-forming units (PFU/ml) of culture supernatant, presented as red circles. **(C)** Cytotoxic effect of i7DMA in infected cells. Vero CCL81 cells were infected with USUV and treated with vehicle (black bar), 7DMA (green bar) or i7DMA (orange bar) at the concentration of 10 µM; non-infected Vero CCL81 cells were treated with 7DMA (green outlined bar) and i7DMA (orange outlined bar) at the same concentration. Cell culture supernatant was collected at 24 h post-infection for the assessment of infectious viral load (green and orange circles for 7DMA and i7DMA, respectively). Cell viability was assessed as described above. Data are representative of two or more independent experiments and indicate mean ±SE (n = 2 to 5).

The 7DMA antiviral activity was tested against the emerging flavivirus USUV (Ashraf et al., 2015) *in vitro*. Vero CCL81 cells, derived from monkey kidney, and SH-SY5Y cells, derived from human neuroblastoma, were infected with USUV and treated with 7DMA (Figures 4A, B). The assessment of infectious viral load in cell culture supernatants indicated that the 7DMA treatment reduced USUV titers at a concentration of 25 µM or greater. The antiviral effect of 7DMA treatment on USUV replication was greater in SH-SY5Y cells than in Vero CCL81 cells, reducing USUV titers by approximately 10,000-fold at a concentration of 100 µM (Figure 4B), with the same treatment condition resulting in approximately 50-fold reduction in the infected Vero CCL81 cells (Figure 4A). Moreover, 7DMA treatment had little effect on the viability of SH-SY5Y cells, as indicated MTT assays, while in Vero CCL81 cells, up to 30% reduction in cell culture viability was observed at the highest concentration tested.

The immediate precursor of 7DMA in this synthetic route, compound **13** (named i7DMA), was also tested against USUV in Vero CCL81 cells (Figure 4C). Although we were not able to differentiate the antiviral effect of 7DMA and i7DMA at the concentration of 10 μM, i7DMA treatment caused a 25% reduction in cell culture viability only in USUV-infected cells. Such an effect on the viability of flavivirus-infected cells was not observed for 7DMA in any of the tested conditions in this study and is not reported in the literature.

## 4 Discussion

Nucleoside analogues are privileged tools in antiviral research. *N*-Glycosylation reactions that unite both sugar and nucleobase moieties commonly follow the Vorbrüggen protocol, using acylated ribosides and nucleobase derivatives, often in solution in acetonitrile, as well as Lewis acids and silylating agents to promote activation of both coupling partners. During the synthesis of the antiviral compound 7-deaza-2′-methyl-adenosine (**2**) applying literature protocols using the Vorbrüggen conditions in the key glycosylation step, we were faced with considerable amounts of a by-product (**10**) and low yields of the desired product (**9**). A full structural characterization, including an X-ray structure of the purified compound led us to conclude that **10** was the product of addition of the solvent, acetonitrile, to activated riboside **11** at its non-anomeric position, a process provoked by the lack of reactivity of the nucleobase and concomitant activation of acetonitrile by the Lewis acid, TMSOTf. To the best of our knowledge, it is the first time that this mechanism is reported, where the solvent acts as competing nucleophile during a Vorbrüggen protocol, and one must notice that this occurrence has never been mentioned by the authors who performed this reaction. No information was given on whether reagents and or solvents were purified prior to use in those reactions. By substituting acetonitrile with 1,2-dichloroethane, the formation of the by-product could be avoided and yields, improved. Overall, this work suggests that for the glycosylation of weakly reactive nucleobases using Vorbrüggen protocols, conditions that preclude acetonitrile as solvent should be chosen, favoring 1,2-dichloroethane, for instance. Alternatively, protocols that make use of already established epoxides could be privileged, although their preparation requires additional synthetic steps. As such, this work proposes a straightforward synthesis of 7DMA (**2**), achievable in three steps from commercially available perbenzoylated 2-methyl-ribose (**5**) and iodopurine (**6**), useful for research projects that demand quantities of 7DMA past the milligram scale, which come at relatively high costs.

7DMA prepared by this route showed moderate activity against the emerging mosquito-borne USUV, in line with previous results (Segura Guerrero et al., 2018). Indeed, other flaviviruses, such as ZIKV and WNV, have already proven more susceptible to 7DMA (Eyer et al., 2016, 2019). Interestingly, 7DMA was less toxic and caused greater reductions in USUV replication when tested in infected SH-SY5Y cells. This cell line is used as a model for neuronal cells, which are considered target cells for USUV in mammalian hosts, especially when USUV infection evolves to neurological disease. There are no antiviral treatments available against USUV infection, and few compounds presenting the antiviral activity against USUV *in vitro* or *in vivo* are reported to date (Segura Guerrero et al., 2018; Caracciolo et al., 2020; Wald et al., 2022; Chen et al., 2023). Altogether, advantages to produce 7DMA using the proposed new route, coupled with preliminary evidence indicating antiviral activity, should facilitate *in vivo* assessment of 7DMA antiviral activities against USUV and other emerging pathogens.

## Data Availability

The datasets presented in this study can be found in online repositories. The names of the repository/repositories and accession number(s) can be found at: https://www.ccdc.cam.ac.uk/structures/, 2220164.
