# Supplementary material for: Competing interests during the key N-glycosylation of 6-chloro-7-deaza-7-iodopurine for the synthesis of 7-deaza-2′-methyladenosine using Vorbrüggen conditions
Source: Front Chem. 2023 Mar 23;11:1163486. doi: 10.3389/fchem.2023.1163486 (PMC10076608; doi:10.3389/fchem.2023.1163486)
Supplement: Supplementary file 1 [file DataSheet1.docx]

Supplementary Material

Competing interests during the key *N*-glycosylation of 6-chloro-7-deaza-7-iodopurine for the synthesis of 7-deaza-2’-methyladenosine (7DMA) under Vorbrüggen conditions

Fabrício Fredo Naciuk^1^, Andrey Fabricio Ziem Nascimento^2^, Rebeca de Paiva Froes Rocha^1^, Joane Kathelen Rustiguel^1^, Lais Durço Coimbra^1^, Rafael Elias Marques^1^, Marjorie Bruder^1*^

^1^Brazilian Biosciences National Laboratory, Brazilian Center for Research in Energy and Materials, Campinas, Sao Paulo, Brazil, ^2^ Brazilian Synchrotron Light Laboratory, Brazilian Center for Research in Energy and Materials, Campinas, Sao Paulo, Brazil

*** Correspondence:** Marjorie Bruder; marjorie.bruder@lnbio.cnpem.br

# Synthetic and Analytical Procedures

## General Information

Reagents and anhydrous solvents were purchased from Merck/Sigma-Aldrich Brasil Ltda for carrying out the reactions, and from Labsynth Produtos para Laboratórios Ltda (analytical grade) for work-up and were used without further purification, unless specified otherwise. 4-Chloro-7H-pyrrolo(2,3-d)pyrimidine 98% was purchased from Oakwood Chemicals. For the *N*-glycosylation step, the glassware was dried at 300 ºC for 16 h and let to cool under an inert atmosphere. Reactions were monitored by thin-layer chromatography (TLC) on Silica gel 60 F254 aluminum sheets and exposed to UV radiation, and/or by treatment with adequate stains and heating. Chromatographic separations were carried out on Merck 60 silica gel (230–400 mesh) in normal phase, and on DSC-C18 solid phase extraction media (18% C loading, 50 µm particle size, 70 Å pore size) in “reverse phase”. Melting points (m.p.) were recorded on a PF 1500 FARMA apparatus with a heating rate of 5 °C min^-1^ and are uncorrected. ^1^H NMR and ^13^C NMR data were recorded on a Varian 500 MHz or Varian 600 MHz spectrometer using as internal standard TMS, or the residual non deuterated solvent. Chemical shifts (δ) were expressed in ppm and multiplicities were reported as singlet (s), broad signal (bs), doublet (d), double doublet (dd), triplet (t), apparent triplet (at), double triplet (dt), quartet (q), double apparent quartet (daq), apparent quartet (aq), quintuplet (quint), multiplet (m), triple triplet (tt). Coupling constants (*J*) are expressed in Hertz (Hz). High-resolution electrospray ionization mass spectrometry (HRMS) was performed on a BRUKER Impact II mass spectrometer, prior to chromatographic separation (Waters BEH C18 column, 50 × 2.1 mm, 1.7 µm) on a Waters Acquity H-Class with PDA detector (190–600 nm), and the data processed using the Bruker Compass Data Analysis 4.3 software (Bruker Daltonics). NMR spectra supporting the synthesis of these compounds are provided in the S1 File. IUPAC names of the compounds were generated using ChemDraw Professional 20.0.

## Synthetic Chemistry

**(2*R*,3*S*,4*R*,5*R*)-2-((Benzyl(methyl)amino)methyl)tetrahydro-2*H*-pyran-2,3,4,5-tetraol (7a)**

D-(+)-Glucose (**7**) (11.2 g, 62.2 mmol) was suspended in ethanol (80 mL) at room temperature. To this mixture was added *N*-methylbenzylamine (8.02 mL, 62.2 mmol, 1.0 equiv.), followed by acetic acid (3.42 mL, 59.7 mmol, 0.96 equiv.). The mixture was then heated at reflux for 3 h and cooled to room temperature. Acetone (100 mL) was added to the thick slurry, and solids were then collected by filtration and washed with acetone. The collected solids were dried under vacuum to give the *title compound* (11.6 g, 40.9 mmol, 66%) was a white solid. HRMS calcd for C_14_H_22_NO_5_^+^ [M+H]^+^: 284.1492; found: 284.1488; ^1^H NMR (500 MHz, DMSO-d_6_) δ 7.36 – 7.28 (m, 4H), 7.28 – 7.21 (m, 1H), 5.30 (s, 1H), 4.48 (d, *J* = 5.7 Hz, 1H), 4.44 (d, *J* = 5.7 Hz, 1H), 4.38 (d, *J* = 3.0 Hz, 1H), 3.80 (d, *J* = 12.0 Hz, 1H), 3.69 – 3.53 (m, 5H), 3.42 (dd, *J* = 12.0, 1.7 Hz, 1H), 2.68 (d, *J* = 13.1 Hz, 1H), 2.53 (d, *J* = 13.1 Hz, 1H), 2.19 (s, 3H); ^13^C NMR (125 MHz, DMSO-d_6_) δ 138.9, 128.8, 128.0, 126.8, 98.0, 69.7, 69.3, 69.0, 63.1, 62.5, 61.8, 43.1.

**(3*R*,4*R*,5*R*)-3,4-Dihydroxy-5-(hydroxymethyl)-3-methyldihydrofuran-2(3*H*)-one (8)**

To a suspension of **7a** (11.6 g, 40.9 mmol) in a mixture of anhydrous methanol (151 mL) and anhydrous THF (50 mL) at room temperature was added anhydrous calcium chloride (1.14 g, 10.2 mmol, 0.25 equiv.), followed by sodium methoxide (25 wt.% solution in methanol, 0.2 mL, 0.86 mmol, 0.22 equiv.). The reaction mixture was then heated to 40 ºC for 19 h. The mixture was actively cooled to 20 ºC in an ice-cooled water bath, upon which temperature Amberlyst®-15 resin (dry) was added (35 g, 300 wt.%). The mixture was then stirred at 20 ºC for 2 h. The resin was removed by filtration and washed with THF. The solvent was removed under vacuum and the residue refluxed in ethyl acetate and then cooled to 0 ºC. The solids were collected by filtration to give the *title compound* (3.5 g, 21.6 mmol, 53%) as a pale grey solid, m.p. 158 – 160 ºC (*lit*.^[[1]](#footnote-1)^ 160 – 161 ºC). ^1^H NMR (500 MHz, DMSO-d_6_) δ 5.97 – 4.62 (m, 3H), 4.18 – 4.13 (m, 1H), 3.78 – 3.67 (m, 2H), 3.51 (dd, *J* = 12.7, 5.0 Hz, 1H), 1.25 (s, 3H); ^13^C NMR (125 MHz, DMSO-d_6_) δ 176.0, 82.6, 71.9, 71.8, 59.4, 20.7.

**(3*R*,4*R*,5*R*)-5-((Benzoyloxy)methyl)-3-methyl-2-oxotetrahydrofuran-3,4-diyl dibenzoate (8a)**

To a suspension of **8** (3.47 g, 21.4 mmol) in anhydrous dichloromethane (80 mL) and triethylamine (14.3 mL, 21.4 mmol, 1.0 equiv.) was added benzoyl chloride (11.2 mL, 21.4 mmol, 1.0 equiv.) dropwise at 0 ºC. The mixture was stirred at ambient temperature for 18 h, after which time methanol (11.2 mL) was added, followed by dichloromethane (80 mL). The mixture was washed with HCl (2M, 4 x 50 mL), brine (1 x 50 mL), dried over MgSO_4_ and concentrated. The residue was purified by crystallization from dichloromethane/isopropanol to yield the *title compound* (8.3 g, 17.5 mmol, 82%) as a white solid, m.p. 159 – 161 ºC (*lit*.^[[2]](#footnote-2)^ 141 – 142 ºC). HRMS calcd for C_27_H_23_O_8_^+^ [M+H]^+^: 475.1387; found: 475.1432; ^1^H NMR (500 MHz, CDCl_3_) δ 8.06 – 8.01 (m, 2H), 7.94 – 7.90 (m, 2H), 7.75 – 7.70 (m, 2H), 7.62 – 7.56 (m, 1H), 7.52 – 7.38 (m, 4H), 7.31 (t, *J* = 7.8 Hz, 2H), 7.17 (t, *J* = 7.8 Hz, 2H), 5.51 (d, *J* = 5.9 Hz, 1H), 5.21 – 5.14 (m, 1H), 4.80 (dd, *J* = 12.5, 3.1 Hz, 1H), 4.69 (dd, *J* = 12.5, 4.1 Hz, 1H), 1.95 (s, 3H); ^13^C NMR (125 MHz, CDCl_3_) δ 172.6, 166.0, 165.9, 165.4, 133.9, 133.7, 133.5, 129.9, 129.7, 129.6, 129.2, 128.6, 128.4, 128.3, 127.8, 127.7, 79.5, 75.3, 72.5, 63.1, 23.6.

**(3*R*,4*R*,5*R*)-5-((Benzoyloxy)methyl)-3-methyltetrahydrofuran-2,3,4-triyl tribenzoate (5)**

To a solution of ribonolactone **8a** (2.55 g, 5.37 mmol) in anhydrous THF (11 mL) was added lithium tri-*t*-butoxyaluminium hydride (LiAl(OtBu)_3_H) (1M, 7.6 mL, 5.37 mmol, 1.4 equiv.) at 0 ºC. The reaction mixture was stirred at room temperature for 3 h (monitored by TLC). The reaction was quenched by addition of NH_4_Cl (10%, 7.6 mL) and of ethyl acetate (7.6 mL). The reaction mixture was filtered, and the aqueous layer was extracted with ethyl acetate (3 x 11 mL). The combined organic layers were dried over MgSO_4_ and evaporated. This residue was then dissolved in anhydrous dichloromethane (23 mL) and Et_3_N (1.2 mL, 8.6 mmol, 1.6 eq), and benzoyl chloride (0.7 mL, 5.91 mmol, 1,1 equiv.) was added dropwise at 0 ºC. The mixture was stirred for 19 h at room temperature, upon which time methanol (0.7 mL) was added, followed by dichloromethane (23 mL). The mixture was washed with HCl (2M, 4 x 23 mL), brine (1 x 23 mL), dried over MgSO_4_ and concentrated. The residue was purified by crystallization from dichloromethane/isopropanol to yield the *title compound* (2.1 g, 3.62 mmol, 67%) as a white solid, m.p. 154 – 156 ºC (*lit*.^2^ 154 – 156.3 ºC). HRMS calcd for C_27_H_23_O_7_^+^ [M-OBz]^+^: 459,1438 and C_34_H_32_NO_9_^+^, 598,2072 [M+NH_4_]^+^, found: 459.1421 and 598.2051; ^1^H NMR (500 MHz, CDCl3) δ 8.14 – 8.10 (m, 4H), 8.08 – 8.04 (m, 2H), 7.92 – 7.87 (m, 2H), 7.65 – 7.58 (m, 3H), 7.52 – 7.39 (m, 7H), 7.18 – 7.13 (m, 2H), 7.07 (s, 1H), 5.95 (d, *J* = 8.1 Hz, 1H), 4.82 – 4.76 (m, 1H), 4.68 (dd, *J* = 12.2, 4.2 Hz, 1H), 4.54 (dd, *J* = 12.2, 4.7 Hz, 1H), 1.95 (s, 3H); ^13^C NMR (125 MHz, CDCl_3_) δ 166.1, 165.6, 164.8, 164.6, 133.8, 133.6, 133.5, 130.3, 133.0, 129.9, 129.9, 129.8, 129.6, 129.4, 129.2, 128.9, 128.6, 128.6, 128.6, 128.2, 97.8, 86.7, 78.6, 76.2, 63.9, 16.9.

**4-Chloro-5-iodo-7*H*-pyrrolo[2,3-*d*]pyrimidine (6)**

A solution of 4-chloro-1*H*-pyrrolo[2,3-*b*]pyridine (**4a**) (3.1 g, 20.0 mmol) was *N*-iodosuccinimide (4.96 g, 22.0 mmol, 1.1 equiv.) in DMF (20 mL) was stirred for 30 minutes. The product was precipitated by the addition of water to yield the *title compound* (5.31 g, 19.0 mmol, 95%) as a brown solid, m.p. 195 – 198 ºC (dec.) (*lit*.^[[3]](#footnote-3)^ 196 – 199 ºC, dec.). HRMS calcd for C_6_H_4_ClIN_3_^+^ [M+H]^+^: 279.9133, found: 279,9145; ^1^H NMR (500 MHz, CDCl_3_) δ 7.96 (s, 1H), 8.61 (s, 1H), 12.94 (s, 1H); ^13^C NMR (125 MHz, DMSO-d_6_) δ 51.6, 115.8, 133.8, 150.5, 150.7, 151.5.

**(2*R*,3*R*,4*R*,5*R*)-5-((Benzoyloxy)methyl)-2-(4-chloro-5-iodo-7*H*-pyrrolo[2,3-*d*]pyrimidin-7-yl)-3-methyltetrahydrofuran-3,4-diyl dibenzoate (9)**

To a mixture of **5** (2.44 g, 4.2 mmol), **6** (1.41 g, 5.04 mmol, 1.2 equiv.), DBU (1.88 ml, 12.6 mmol, 3.0 equiv.) and molecular sieves 3 Å (1.68 g) in 1,2-dichloroethane (42 ml), TMSOTf (3.04 ml, 16.8 mmol, 4.0 equiv.) was added dropwise at 0 ºC and the mixture was then stirred at 70 ºC for 24 h. After cooling, the mixture was diluted with ethyl acetate (42 ml) and saturated aq. NaHCO_3_ (42 ml) was added. The aqueous layer was further extracted with ethyl acetate (4 x 42 mL). The combined organic layers were washed with water (42 ml) and brine (42 ml), dried over MgSO_4_ and concentrated. The crude product was purified once by chromatography on silica gel (CHCl_3_/MeOH, 98:2) and once by reverse phase chromatography (MeCN/H_2_O, 80:20) to yield the *title compound* (1.81 g, 2.45 mmol, 58%) as white solid, m.p. 96 – 98 ºC (*lit*.^[[4]](#footnote-4)^ 95 – 97 ºC). HRMS calcd for C_33_H_26_ClIN_3_O_7_^+^ [M+H]^+^: 738,0498, found: 738.0496; ^1^H NMR (500 MHz, CDCl_3_) δ 8.74 (s, 1H), 8.13 – 8.08 (m ,4H), 7.97 – 794 (m , 2H), 7.69 (s, 1H), 7.64 – 7.51 (m, 3H), 7,49 – 7.43 (m, 4H), 7.34 (apt, *J* = 8.08 Hz, 2H), 6.95 (s, 1H), 6.03 (d, *J* = 5.9 Hz, 1H), 4.95 (dd, *J* = 12.3, 3.4 Hz, 1H), 4.85 (dd, *J* = 12.3, 5.8 Hz, 1H), 4.75 – 4.69 (m, 1H), 1.59 (s, 3H); ^13^C NMR (125 MHz, DMSO-d_6_) δ 166.3, 165.3, 165.1, 153.2, 151.2, 150.7, 133.7, 133.7, 133.4, 133.0, 130.0, 129.8, 129.7, 129.6, 129.5, 128.7, 128.6, 128.5, 128.5, 117.7, 89.0, 85.0, 80.0, 75.6, 63.3, 52.7, 18.0.

**(2*R*,3*R*,4*R*,5*R*)-2-(4-Amino-5-iodo-7*H*-pyrrolo[2,3-*d*]pyrimidin-7-yl)-5-(hydroxymethyl)-3-methyltetrahydrofuran-3,4-diol (13)**

A mixture of compound **9** (935 mg, 1.29 mmol) and aq. ammonia (28% w/w, 27 mL) in dioxane (27 ml) was stirred in a sealed tube at 120 ºC for 23 h. After cooling, the volatiles were evaporated and the crude product was purified by chromatography on silica (CHCl_3_/MeOH, 85:15) to yield the *title compound* (503 mg, 1.24 mmol, 96%) as brownish solid, m.p. 204 – 207 ºC (*lit*.^4^ 207 – 208 ºC). HRMS calcd for C_12_H_16_IN_4_O_4_^+^ [M+H]^+^: 407.0211, found: 407.0207; ^1^H NMR (500 MHz, CD_3_OD) δ 8.10 (s, 1H), 7.79 (s, 1H), 6.23 (s, 1H), 4.12 – 4.07 (m, 1H), 4.04 – 3,97 (m, 2H), 3.83 (dd, *J* = 12.4, 2.8 Hz, 1H), 0.83 (s, 3H); ^13^C NMR (125 MHz, CD_3_OD) δ 159.0; 153.0; 151.0; 128.8; 105.2; 93.0; 84.0; 80.6; 73.6; 61.1; 51.3; 20.1.

**(2*R*,3*R*,4*R*,5*R*)-2-(4-Amino-7H-pyrrolo[2,3-*d*]pyrimidin-7-yl)-5-(hydroxymethyl)-3-methyltetrahydrofuran-3,4-diol, 7DMA (2)**

A solution of compound **13** (1.43 g, 3.53 mmol) and Et_3_N (0.36 mL, 3.53 mmol, 1.0 equiv.) in a (60:1) MeOH/DMF mixture (50 mM; 71 mL) was passed through the H-Cube® Pro reactor, equipped with a 30 mm Pd/C 10% cartridge, in “full H_2_ mode” (<10 bar), at 40 ºC, at a 1 mL/min of flow. After removing the solvent, the crude product was purified once by chromatography on silica gel (EtOH/H_2_O/NH_4_OH – 9.8:0.1:0.1) and again eluting with THF (100%) to yield the *title compound* (0.76 g, 2.71 mmol, 77%) as a white solid m.p. 218 – 220 ºC (*lit*.^[[5]](#footnote-5)^ 222 ºC). HRMS calcd for C_12_H_17_N_4_O_4_^+^ [M+H]^+^: 281.1244, found: 281.1244; ^1^H NMR (500 MHz, CD_3_OD) δ 8.08 (s, 1H), 7.48 (d, *J* = 3,7 Hz, 1H), 6.60 (d, *J* = 3,7 Hz, 1H), 6.22 (s, 1H), 4.12 (d, *J* = 8.9 HZ, 1H), 4.05 – 3.99 (m, 2H), 3.84 (dd, *J* = 13,0, 3,7 Hz, 1H), 0.81 (s, 3H); ^13^C NMR (125 MHz, CD_3_OD) δ 159.2, 152.3, 150.7, 123.7, 104.8, 100.8, 93.4, 83.9, 80.6, 74.1, 61.6, 20.1.

## NMR Analysis Interpretation for compound 10

Compound **10** was subjected to a series of NMR experiments, starting with one-dimensional ^1^H and ^13^C NMR experiments. We first looked at potential perbenzoylated ribose signal patterns and could indeed observe aromatic peaks between 7.97 and 7.31 ppm integrating for 15 hydrogens, as expected. The sugar backbone was also evidenced by the presence of a singlet at 5.88 ppm integrating for one hydrogen attributed to the anomeric H1. The H5a and H5b hydrogens were assigned as double doublets at 4.37 ppm (*^2^J*_geminal_ = 12.39 Hz and ^3^*J*_H5a-H4_ = 3.40 Hz) and at 4.17 ppm (^2^*J*_geminal_ = 12.45 Hz and ^3^*J*_H5b-H4_ = 4.99 Hz), respectively. In addition to the coupling constants, assignments were also made based on correlations observed in 2D spectra, such as COSY and NOESY. A doublet (^3^*J*_H3-H4_ = 8.95 Hz) integrating for one hydrogen at 5.12 ppm attributed to H3, as it neighbors the quaternary C2, thus only coupling with adjacent H4, the latter signal being attributed to a multiplet integrating for one hydrogen at 3.65-3.67 ppm. The C2 methyl group appeared clearly as a singlet integrating for three protons at 1.88 ppm. With all the expected signals identified for the riboside moiety, there was one last doublet located at 2.91 ppm and integrating for two hydrogens (denominated H6), which were assigned as H6a and H6b hydrogens with a geminal coupling constant of 4.57 Hz.

The ^13^C NMR spectra were quite consistent with a carbohydrate structure, in addition to two additional signals at 33.8 ppm matching a methylene group, and a quaternary carbon at 115.9 ppm that could potentially be attributed to a nitrile group, supporting our hypothesis that the by-product might be the result of a reaction between activated riboside **11** and acetonitrile. A few inconsistencies were however observed, one being the lack of one of the three ester carbonyls. Conversely, a signal for a quaternary carbon at 111.6 ppm was present in the spectrum, which given its relatively highly de-shielded nature, should imply it is bound to a highly electron-withdrawing element. Given that the most electron-withdrawing atoms present in our proposed by-product are oxygens, one could conclude that this carbon is in fact a ketal. Further, in the aromatic region between 120 and 150 ppm, a peak was substantially de-shielded in relation to the others, at 140.0 ppm, and being of quaternary nature, it could correspond to an alkylated aromatic carbon.

Moving to two-dimensional NMR experiments, special attention was naturally given to the “new” H6 methylene group. HSQC experiments correlated H6 to the methylene carbon at 33.8 ppm, suggesting it is adjacent to an electron-withdrawing group. HMBC experiments revealed that H6 strongly correlated with two quaternary carbons at 111.6 ppm (ketal), 115.9 ppm (nitrile), for one could propose that H6 is adjacent to a nitrile group. H6 also correlated with the aromatic carbon at 140.0 ppm and NOESY experiments further showed that H6 correlated with an aromatic proton. As such, H6 could be proposed to occupy a homobenzylic position, being sandwiched between a nitrile and a ketal group. Regarding the assumed ketal carbon, the latter was also found to correlate with an aromatic proton, and to some extent with the anomeric proton (H1) in HMBC experiments, suggesting it may occupy the benzylic position (next to the carbon at 140.0 ppm), and form the ketal with the C1 oxygen of the riboside. Finally, the NOESY spectra showing strong correlations between protons H1, H3 and the methyl group at C2, and given that the latter two are known to be positioned on the β-face of the ribose ring, one could conclude that the anomeric proton also adopts a β-conformation.

# Single Crystal X-ray Studies of compound 10

Data for the crystal structure was deposited at the CCDC under the deposition number 2220164.

**Supplementary Table 1**

Crystal data and refinement details for compound **10** compound.

|  | compound **10** |
| --- | --- |
| Molecular formula | C_27_H_25_NO_7_ |
| Mr | 499.50 |
| Crystal system, space group | Monoclinic, P2_1_ |
| a, b, c (Å) | 10.4700(10), 10.6100(3), 12.0800(5) |
| α, β, γ (°) | 90, 110.372(5), 90 |
| V (Å^3^) | 1257.99(14) |
| Z | 2 |
| Radiation source, wavelength (Å) | Synchrotron, 0.67937 |
| μ (mm^-1^) | 0.086 |
| Crystal size (mm) | 0.400 x 0.110 x 0.025 |
| T_min_, T_max_ | 0.9, 1.0 |
| No. of measured, independent and observed [I>2.0σ(I)] reflections | 105181, 6913, 6870 |
| (sinθ/λ)_max_ (Å^-1^) | 0.705 |
| R [*F^2^* > 2σ(*F^2^*)], wR(*F^2^*), S | 0.029, 0.076, 1.056 |
| No. of reflections | 6909 |
| No. of parameters | 336 |
| Δρ_max_, Δρ_min_ (eÅ^3^) | 0.30, -0.20 |
| CCDC number | 2220164 |

**Supplementary Table 2**

Hydrogen-bond geometry and non-bonding contacts (Å, °) for compound **10**.

| D-H···*^A^* | D-H | H···*^A^* | D···*^A^* | D-H···*^A^* |
| --- | --- | --- | --- | --- |
| C(1)-H(1)...O(2) | 1.000 | 2.519 | 3.266 | 131.20 |
| C(3)-H(3)...N(1) | 1.000 | 2.523 | 3.354 | 140.34 |


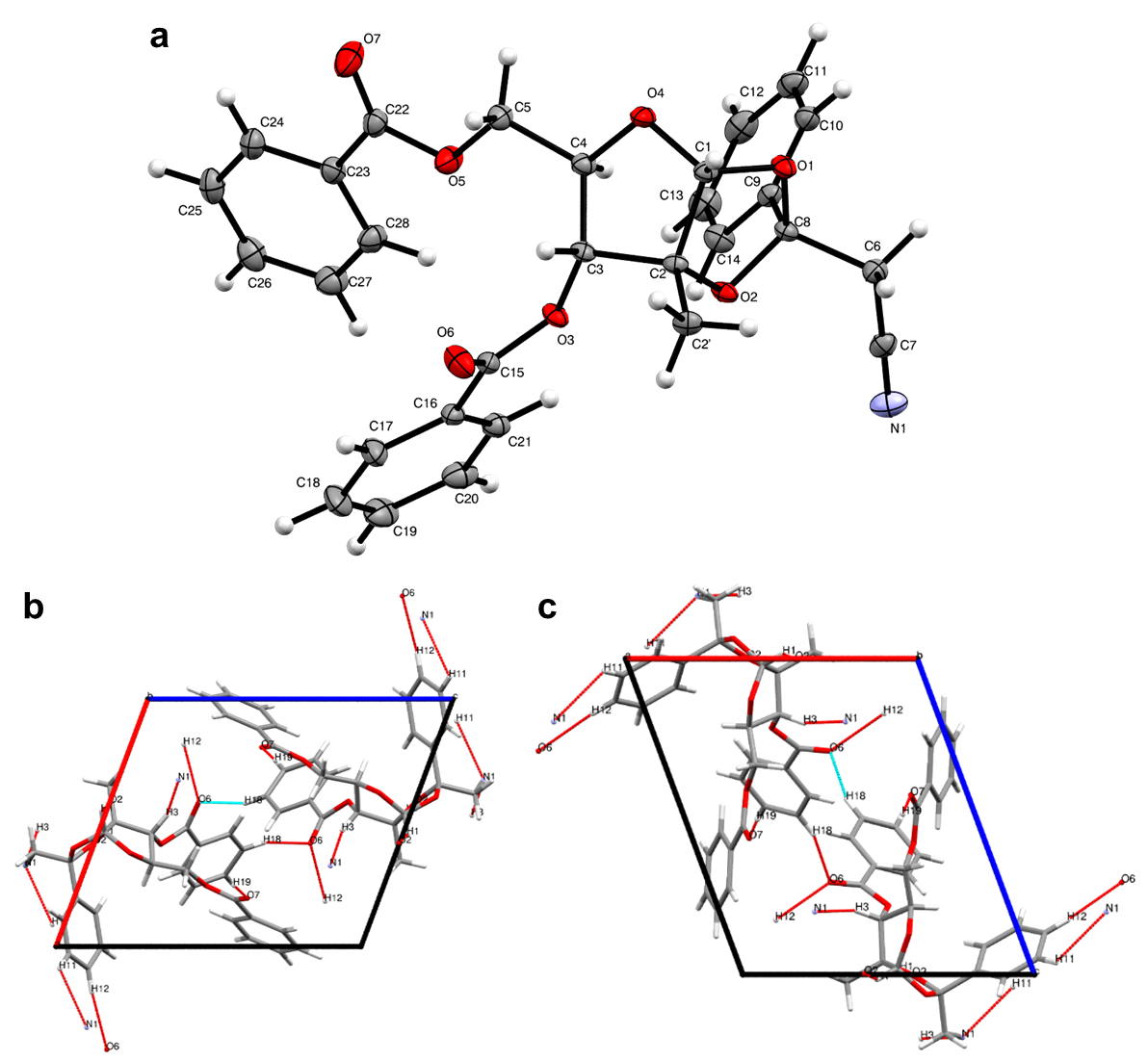


**Supplementary Figure 1.** (a) An ORTEP style view of the X-ray crystal structure of compound **10** showing the atom-numbering scheme. Displacement ellipsoids are drawn at the 50% probability level and H atoms are shown as small spheres of arbitrary radii. (b-c) Packing diagrams for compound **10**, showing the supramolecular assembly and the H-bonds as dashed lines.

# UPLC-MS analyses of the *N*-glycosylation reaction under different conditions.

For each reaction (~0.2 mmol of **5**), aliquots were taken at time points t = 0, 0.5, 1, 6 and 25 h, t = 0 being the time at which all reagents had been added to the reaction mixture. These aliquots were then injected in a Waters Acquity H-Class with PDA detector (190–600 nm) hyphenated to a BRUKER Impact II mass spectrometer in (positive mode). The [M+H]^+^ values for the starting material (**5**), products (**9** and **14**) and the potential by-product (**10**) were monitored (Supplementary Figure 2).


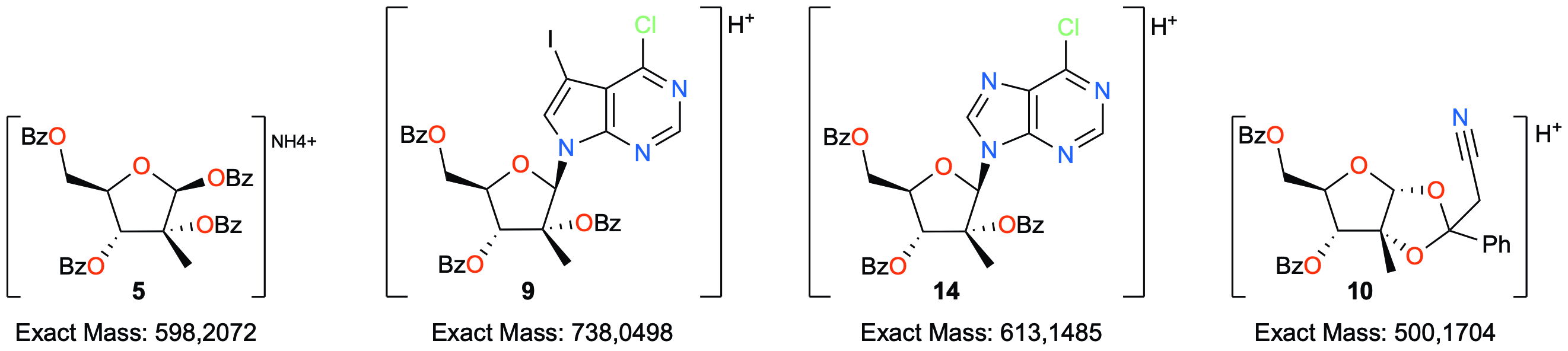


| **Time (h)** | **Method** | **Nucleobase** | **Solvent** | **Relative peak areas (%) by UPLC-MS/UV analyses** | | |
| --- | --- | --- | --- | --- | --- | --- |
|  |  |  |  | **5** | **9/14*** | **10** |
| 0 | A | **6** | MeCN | **100** | 0 | 0 |
| 0.5 | A | **6** | MeCN | **100** | 0 | 0 |
| 1 | A | **6** | MeCN | **100** | 0 | 0 |
| 6 | A | **6** | MeCN | 2 | **68** | **30** |
| 25 | A | **6** | MeCN | 0 | **70** | **30** |
| 0 | B | **6** | MeCN | **100** | 0 | 0 |
| 0.5 | B | **6** | MeCN | **100** | 0 | 0 |
| 1 | B | **6** | MeCN | 2 | **14** | **84** |
| 6 | B | **6** | MeCN | 0 | **11** | **89** |
| 25 | B | **6** | MeCN |  |  |  |
| 0 | A | **6** | DCE | **100** | 0 | 0 |
| 0.5 | A | **6** | DCE | **100** | 0 | 0 |
| 1 | A | **6** | DCE | **100** | 0 | 0 |
| 6 | A | **6** | DCE | **18** | **82** | 0 |
| 25 | A | **6** | DCE | **7** | **93** | 0 |
| 0 | A | **6#** | MeCN | **100** | 0* | 0 |
| 0.5 | A | **6#** | MeCN | 1 | **99*** | 0 |
| 1 | A | **6#** | MeCN | 6 | 94* | 0 |
| 6 | A | **6#** | MeCN | 60 | 40* | 0 |
| 25 | A | **6#** | MeCN | 0 | 0* | 0 |

**Supplementary Figure 2.** Structures, exact mass and relative peak areas of monitored species under different reaction conditions. Method A: TMSOTf/DBU, 70 ºC; Method B: TMSOTf/BSA, 80 ºC.

^a^ 6-Chloropurine (**6#**); DCE, 1,2-dichloroethane; MeCN, acetonitrile.

# NMR Spectra of compounds 7a, 8, 8a, 5, 6, 9, 13, 2 and 10

**
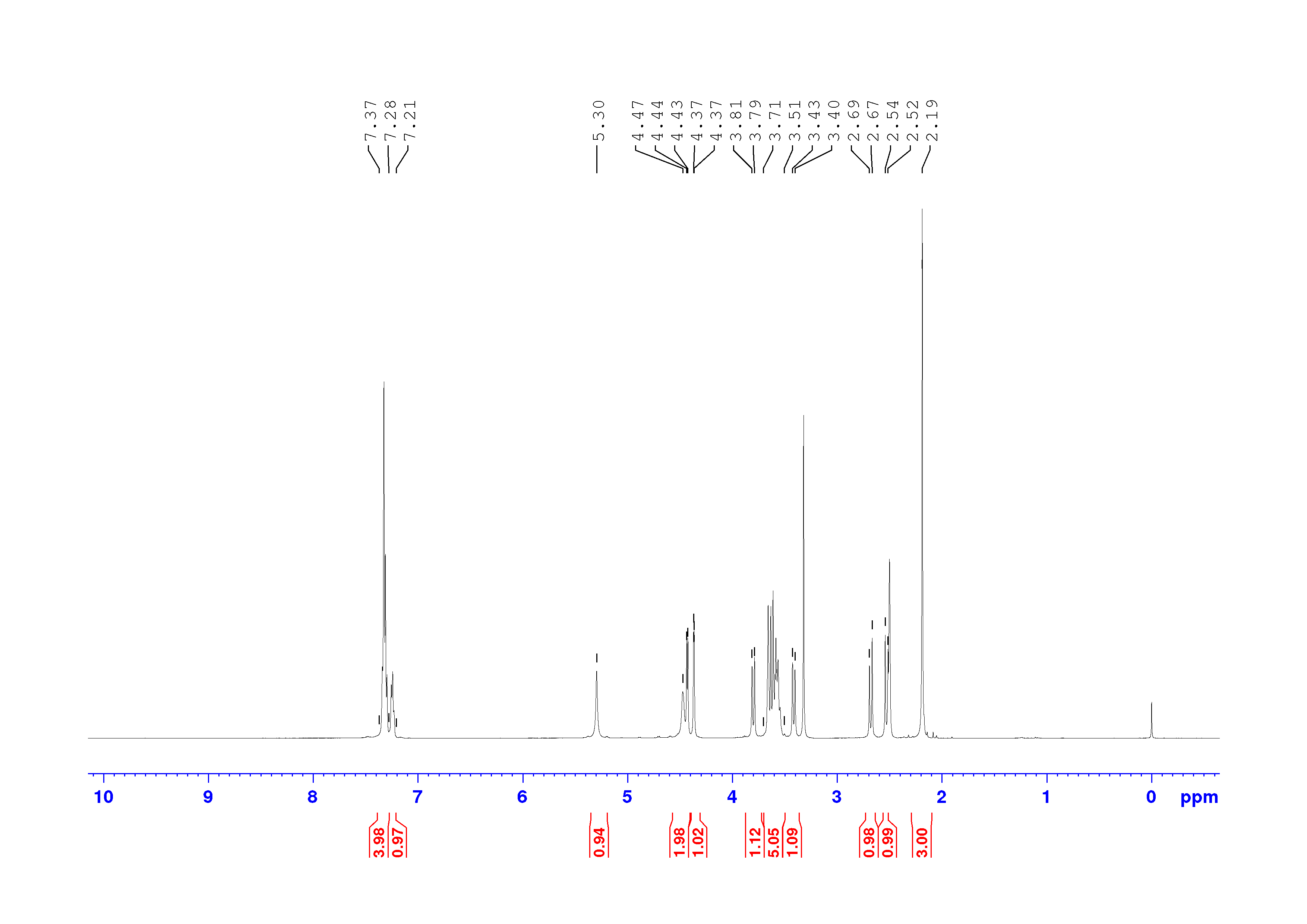
**

**(2*R*,3*S*,4*R*,5*R*)-2-((Benzyl(methyl)amino)methyl)tetrahydro-2*H*-pyran-2,3,4,5-tetraol (7a) – 1H – DMSO-d6 – 500 MHz**

**(2*R*,3*S*,4*R*,5*R*)-2-((Benzyl(methyl)amino)methyl)tetrahydro-2*H*-pyran-2,3,4,5-tetraol (7a) – 13C – DMSO-d6 – 125 MHz
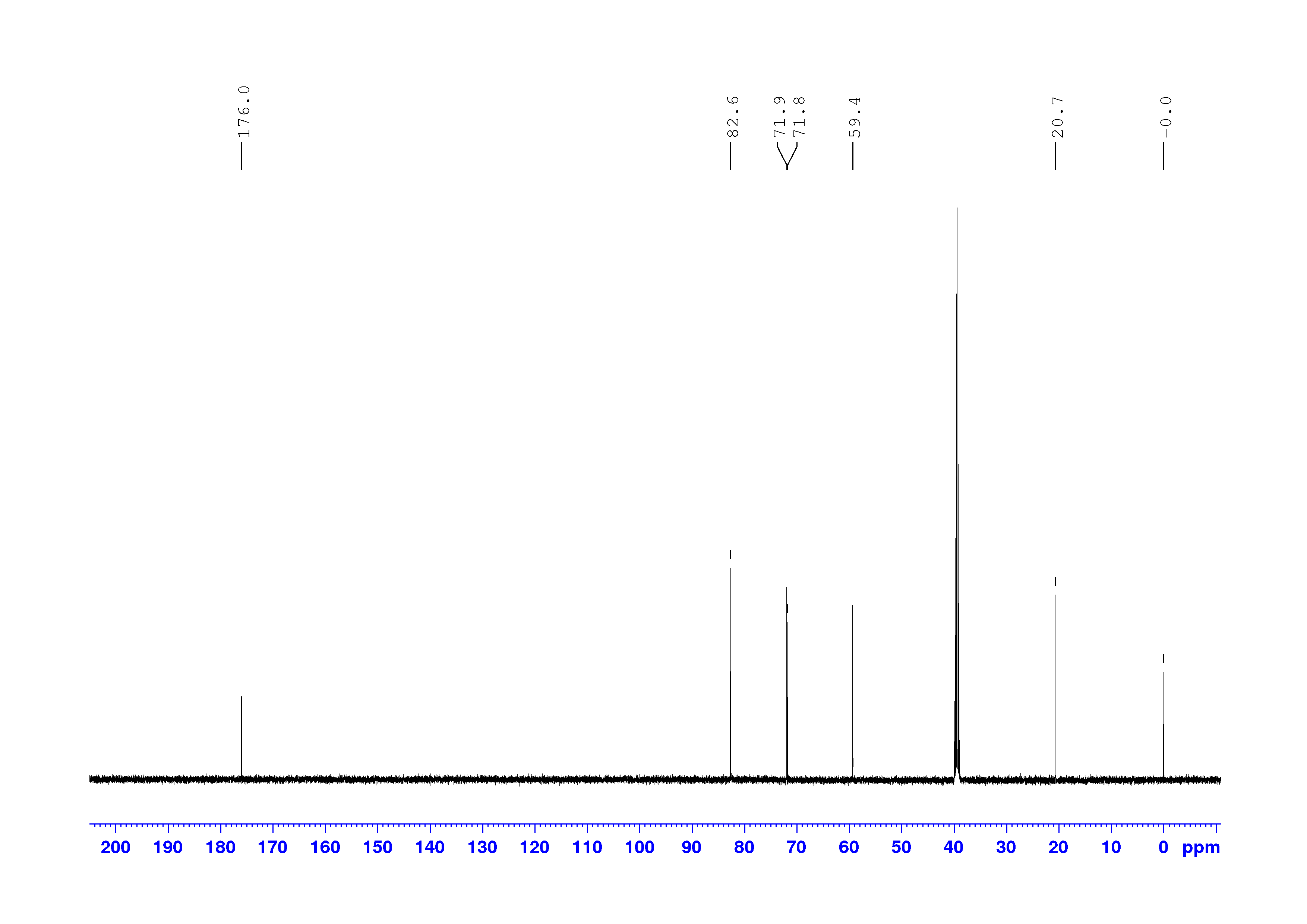
**

**
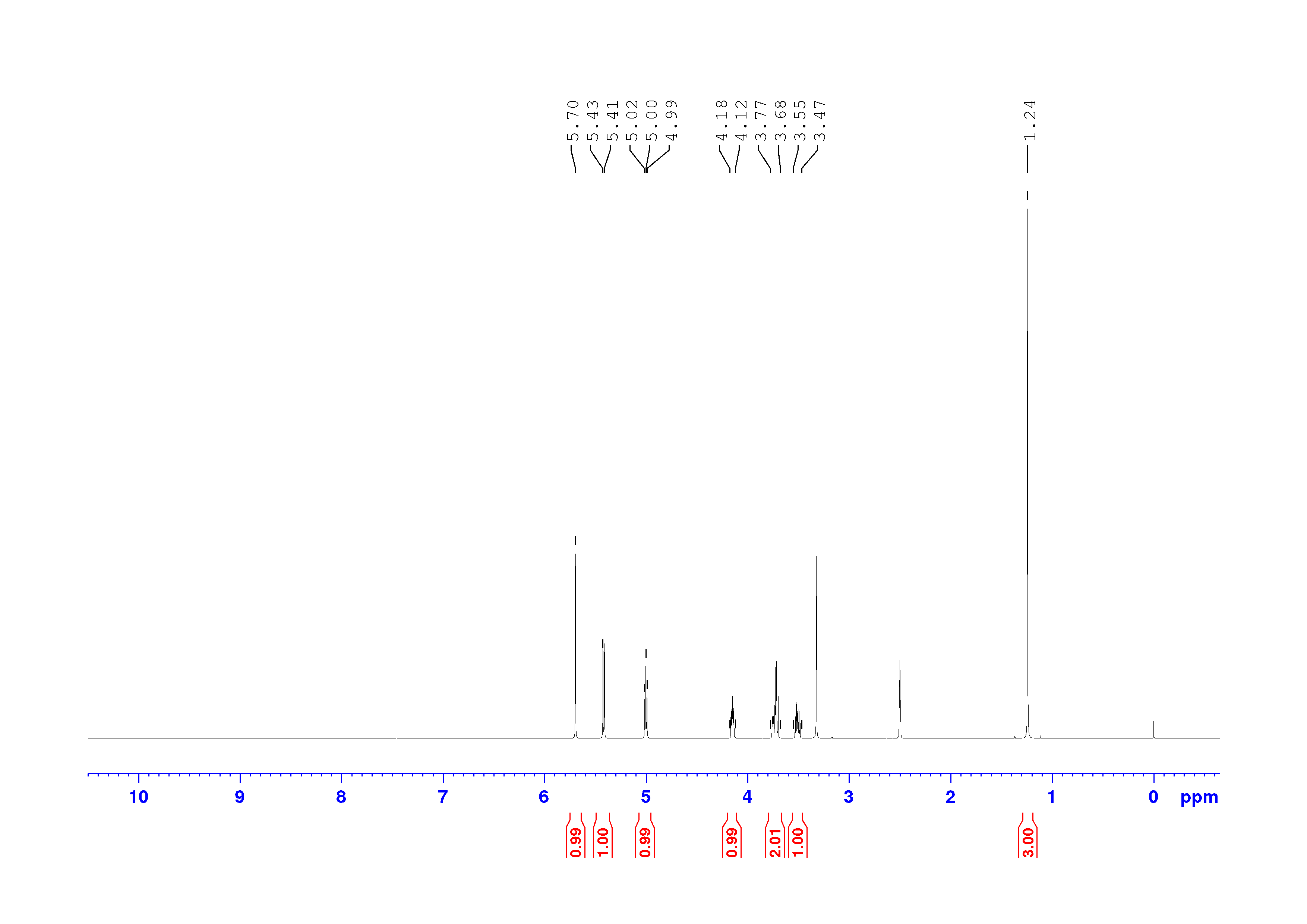
(3*R*,4*R*,5*R*)-3,4-Dihydroxy-5-(hydroxymethyl)-3-methyldihydrofuran-2(3*H*)-one (8) – 1H – DMSO-d6 – 500 MHz**

**
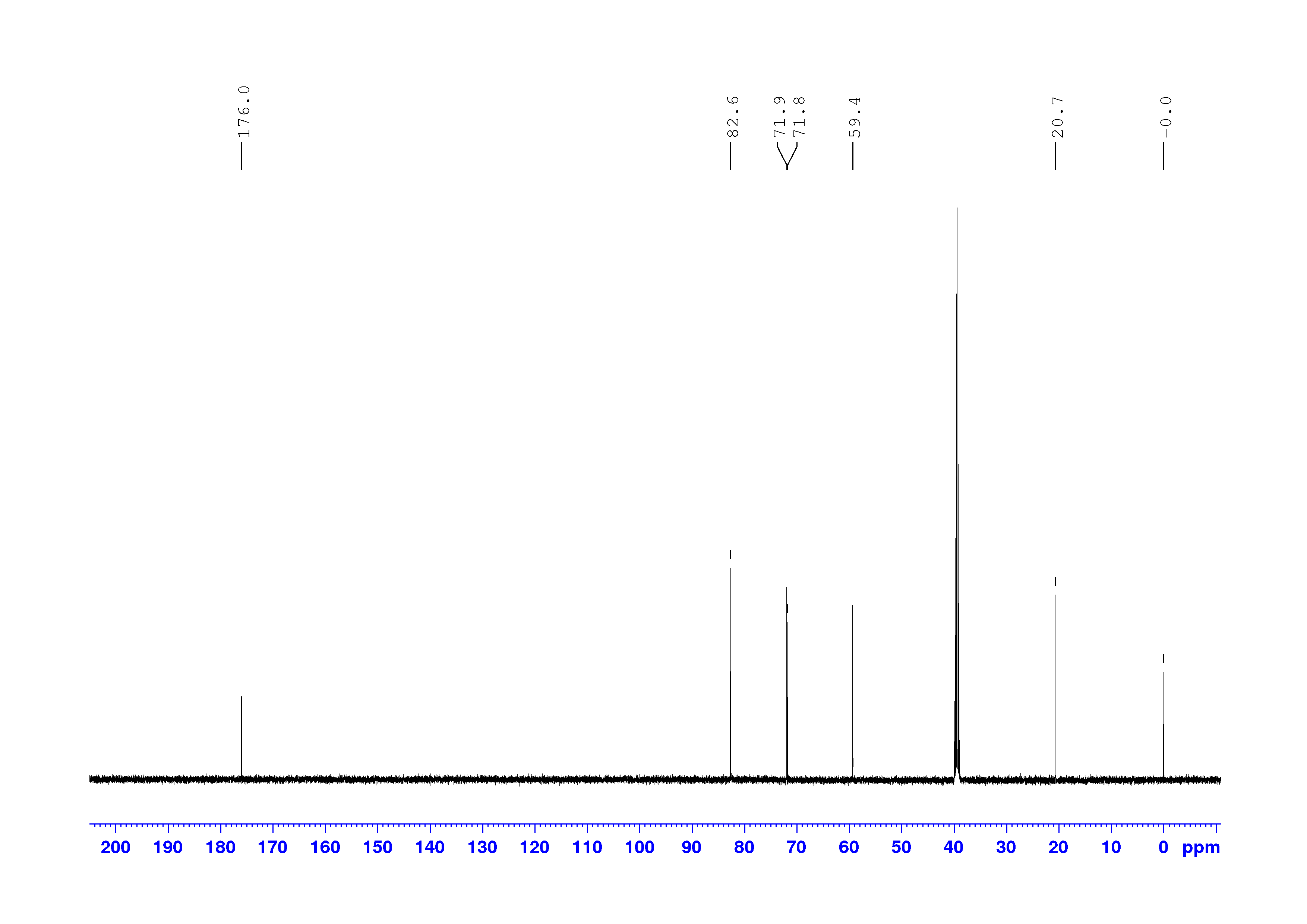
(3*R*,4*R*,5*R*)-3,4-Dihydroxy-5-(hydroxymethyl)-3-methyldihydrofuran-2(3*H*)-one (8) – 13C – DMSO-d6 – 125 MHz**

**
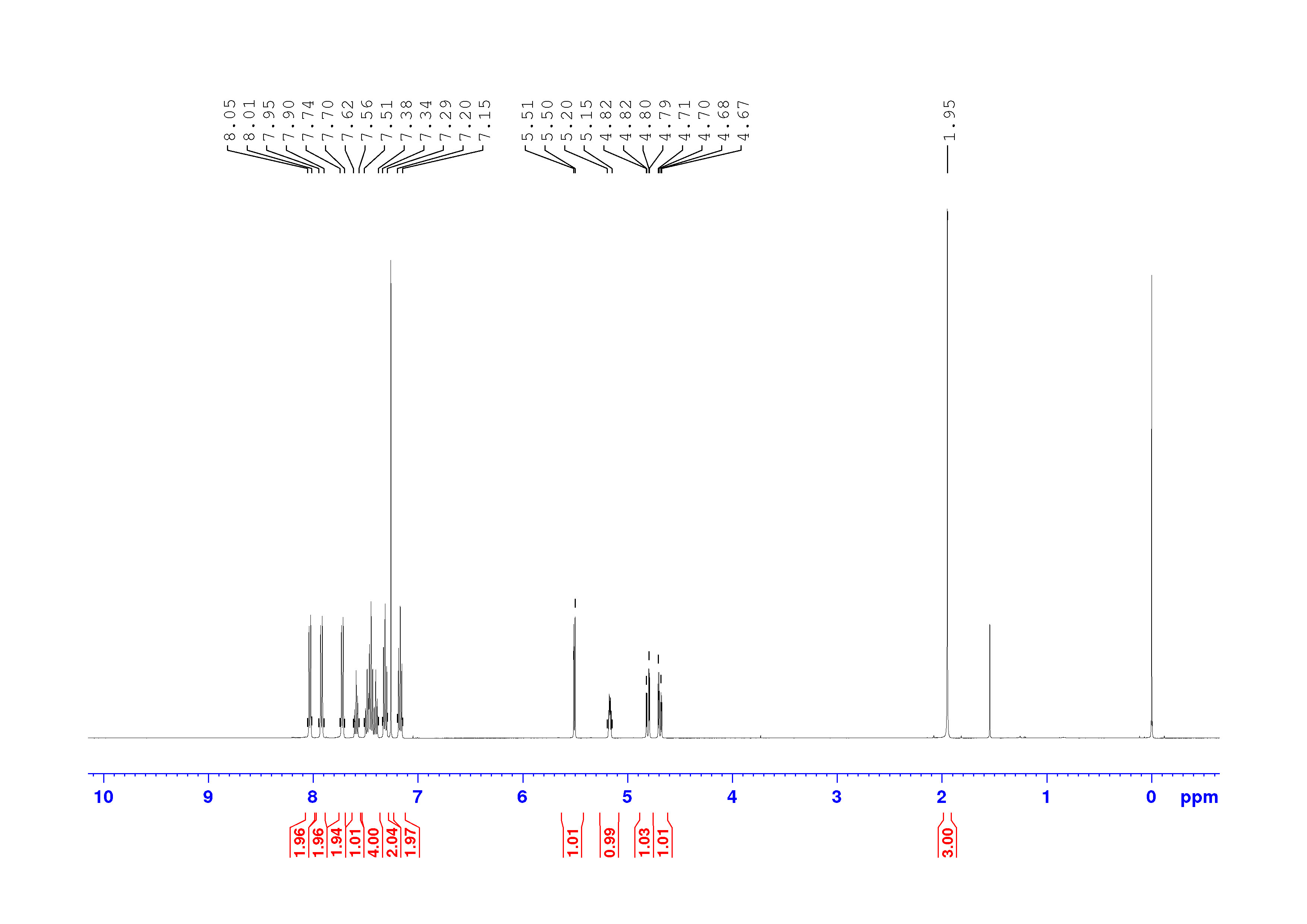
(3*R*,4*R*,5*R*)-5-((Benzoyloxy)methyl)-3-methyl-2-oxotetrahydrofuran-3,4-diyl dibenzoate (8a) – 1H – CDCl_3_ – 500 MHz**

**
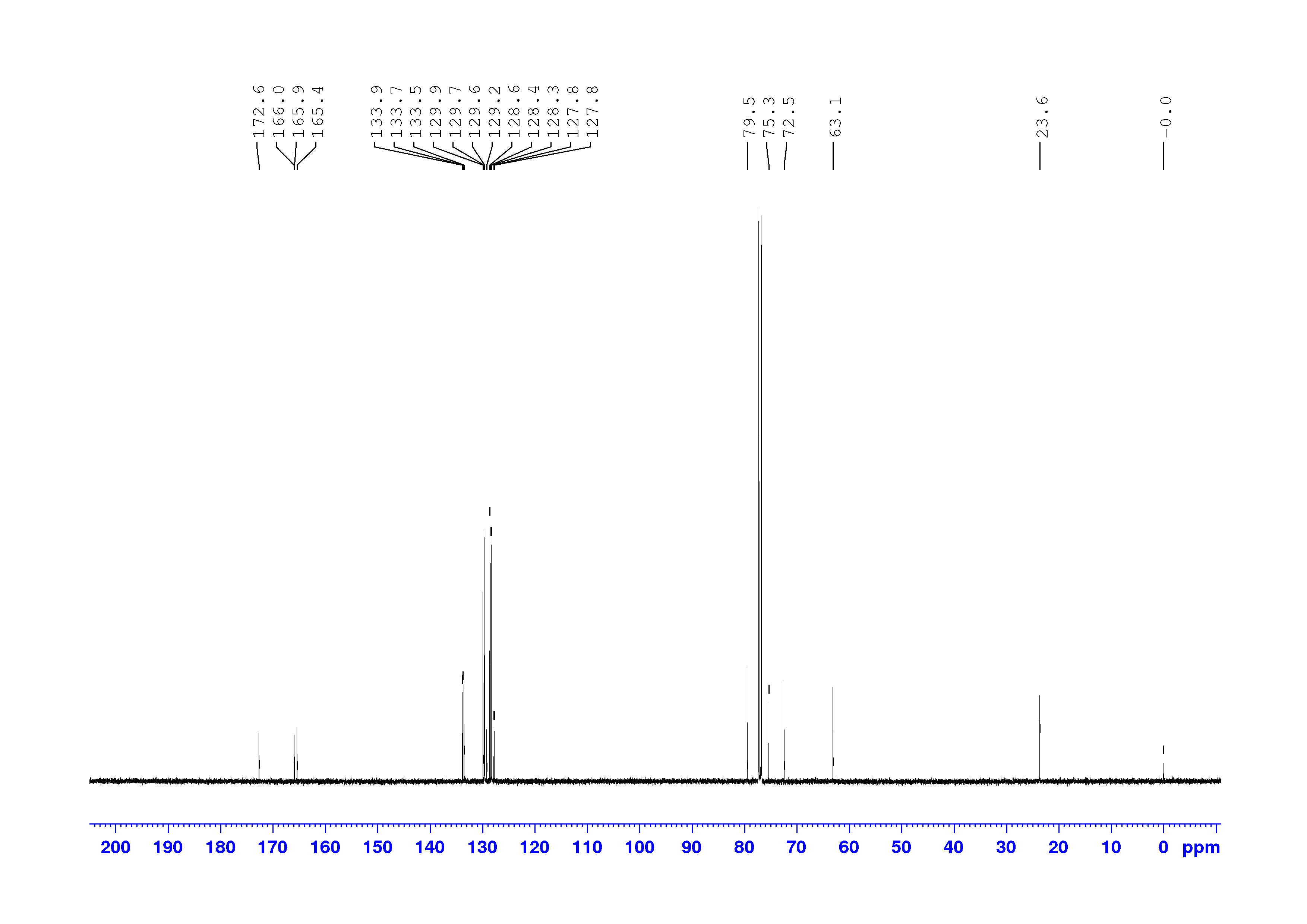
(3*R*,4*R*,5*R*)-5-((Benzoyloxy)methyl)-3-methyl-2-oxotetrahydrofuran-3,4-diyl dibenzoate (8a) – 13C – CDCl_3_ – 125 MHz**

**
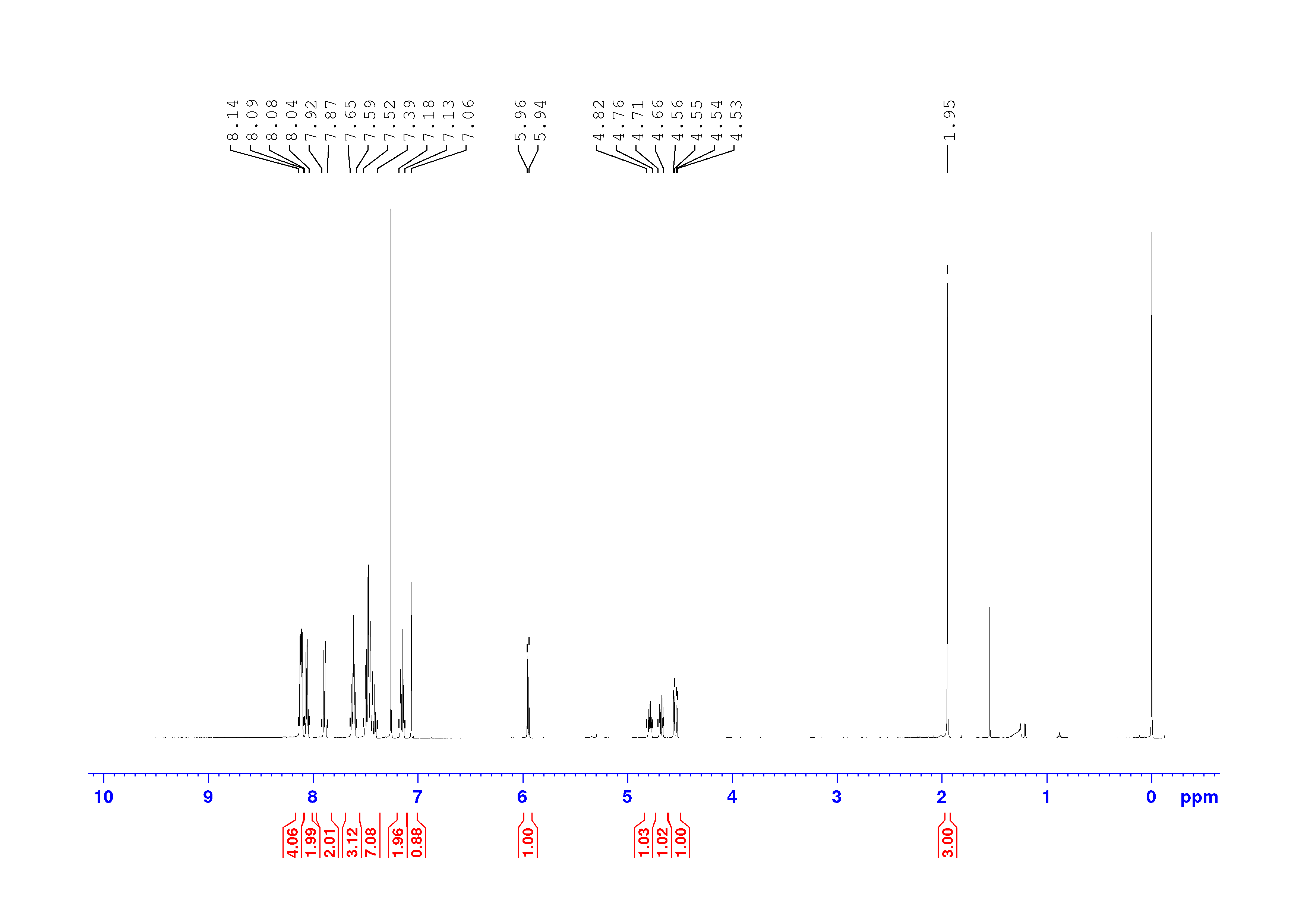
(3*R*,4*R*,5*R*)-5-((Benzoyloxy)methyl)-3-methyltetrahydrofuran-2,3,4-triyl tribenzoate (5) – 1H – CDCl_3_ – 500 MHz**

**
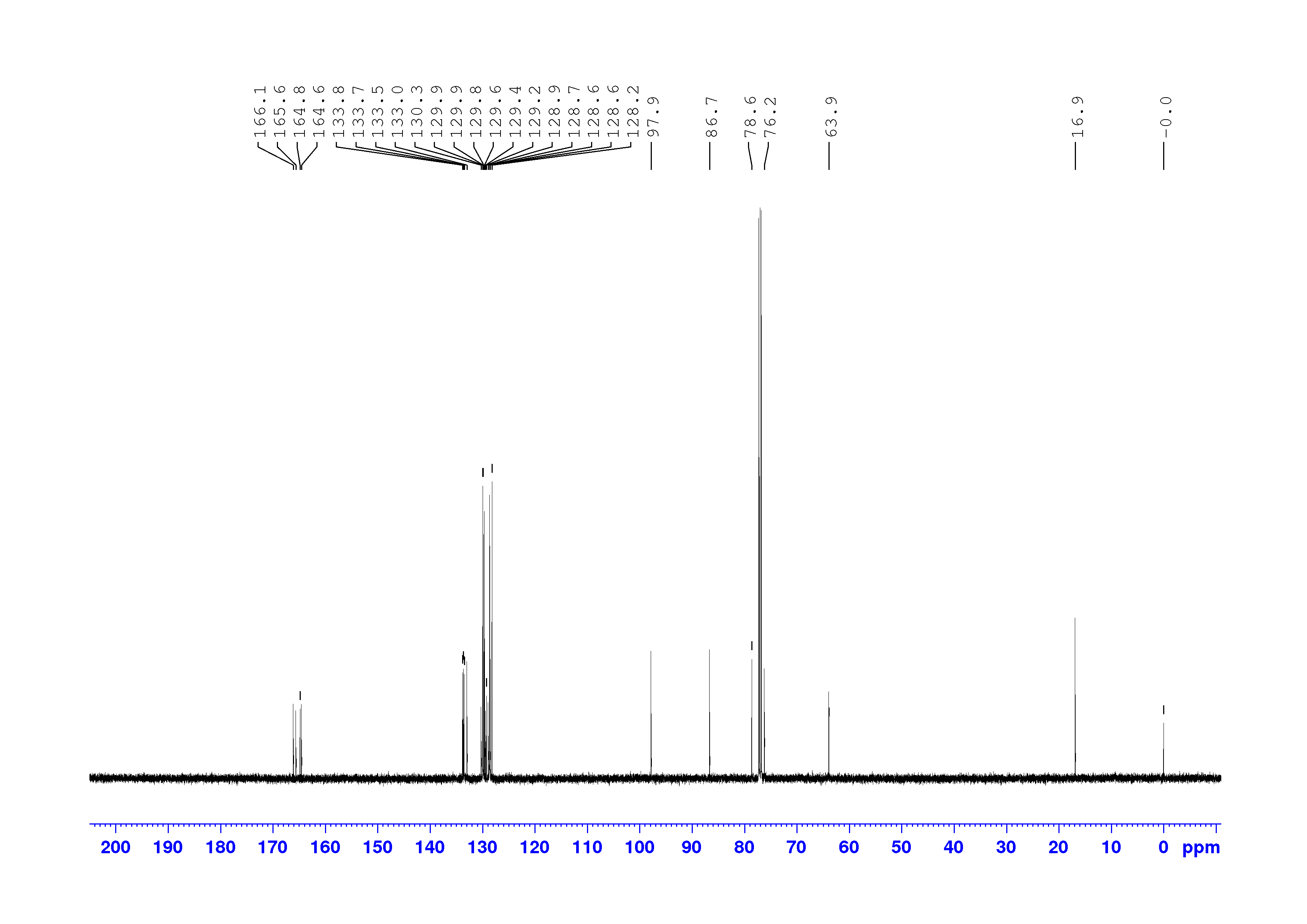
(3*R*,4*R*,5*R*)-5-((Benzoyloxy)methyl)-3-methyltetrahydrofuran-2,3,4-triyl tribenzoate (5) – 13C – CDCl_3_ – 125 MHz**

**
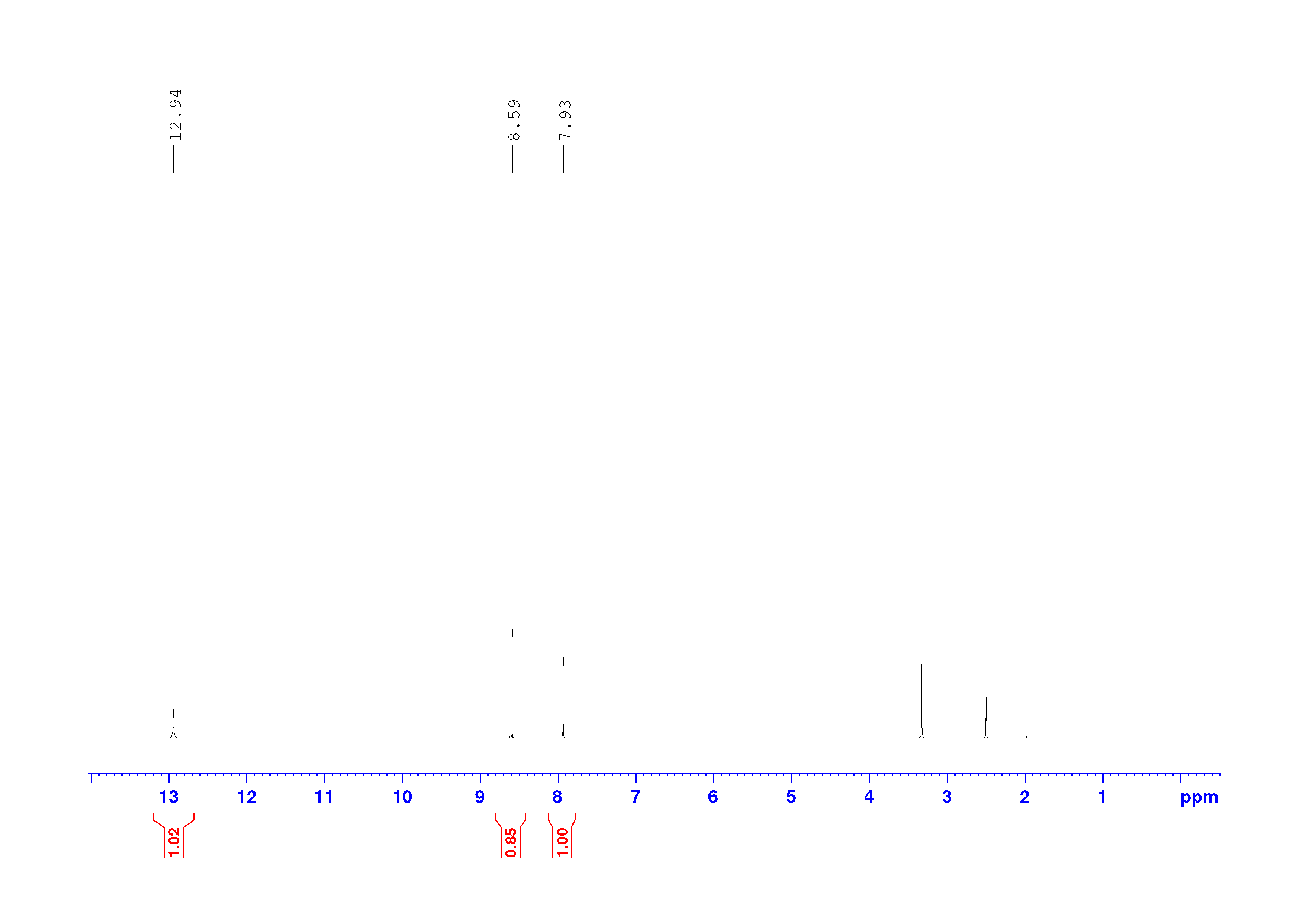
**

**4-Chloro-5-iodo-7*H*-pyrrolo[2,3-*d*]pyrimidine (6) – 1H – CDCl_3_ – 500 MHz**

**
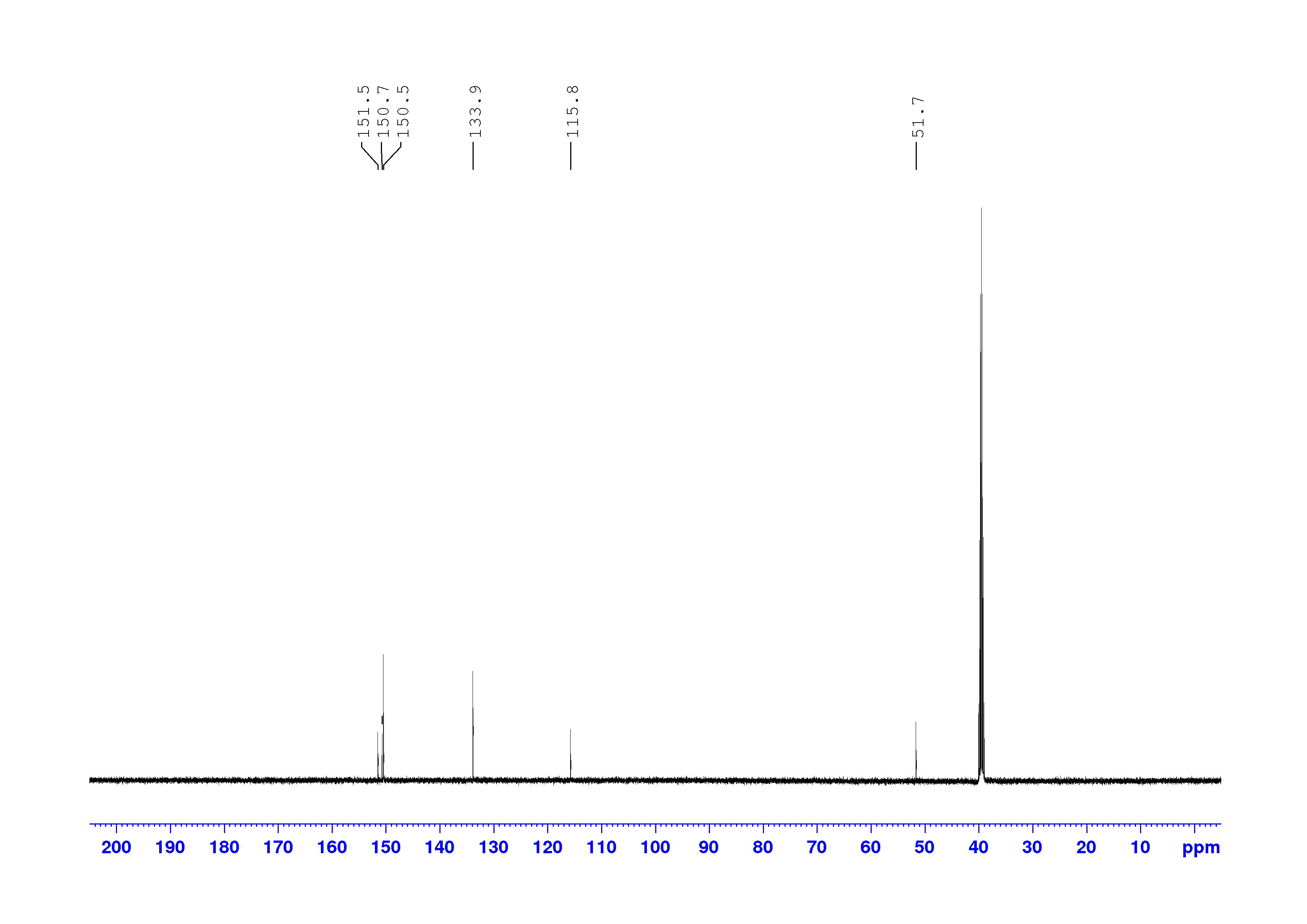
**

**4-Chloro-5-iodo-7*H*-pyrrolo[2,3-*d*]pyrimidine (6) – 13C – CDCl_3_ – 125 MHz**

**
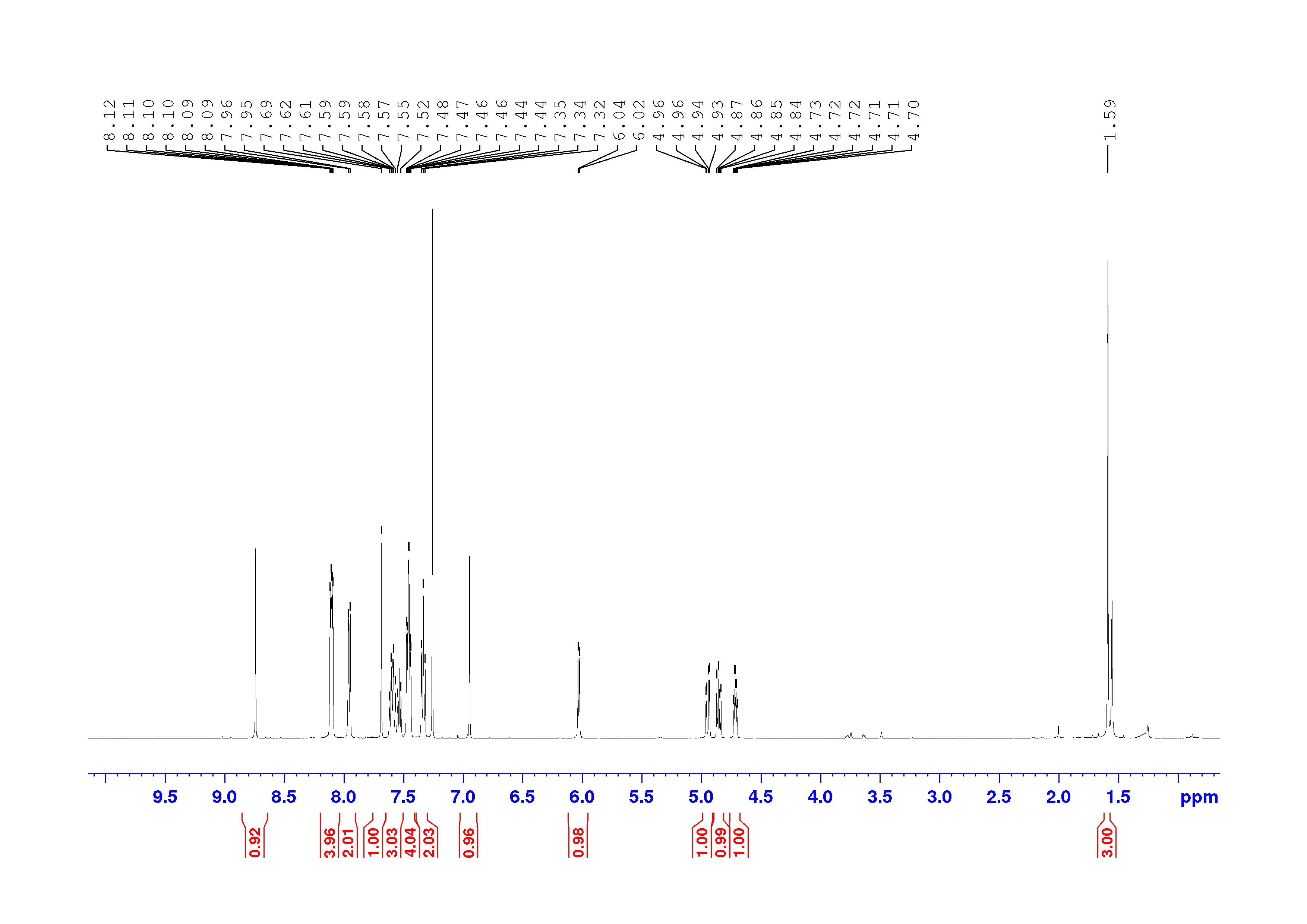
**

**(2*R*,3*R*,4*R*,5*R*)-5-((Benzoyloxy)methyl)-2-(4-chloro-5-iodo-7*H*-pyrrolo[2,3-*d*]pyrimidin-7-yl)-3-methyltetrahydrofuran-3,4-diyl dibenzoate (9) – 1H – CDCl_3_ – 500 MHz**

**
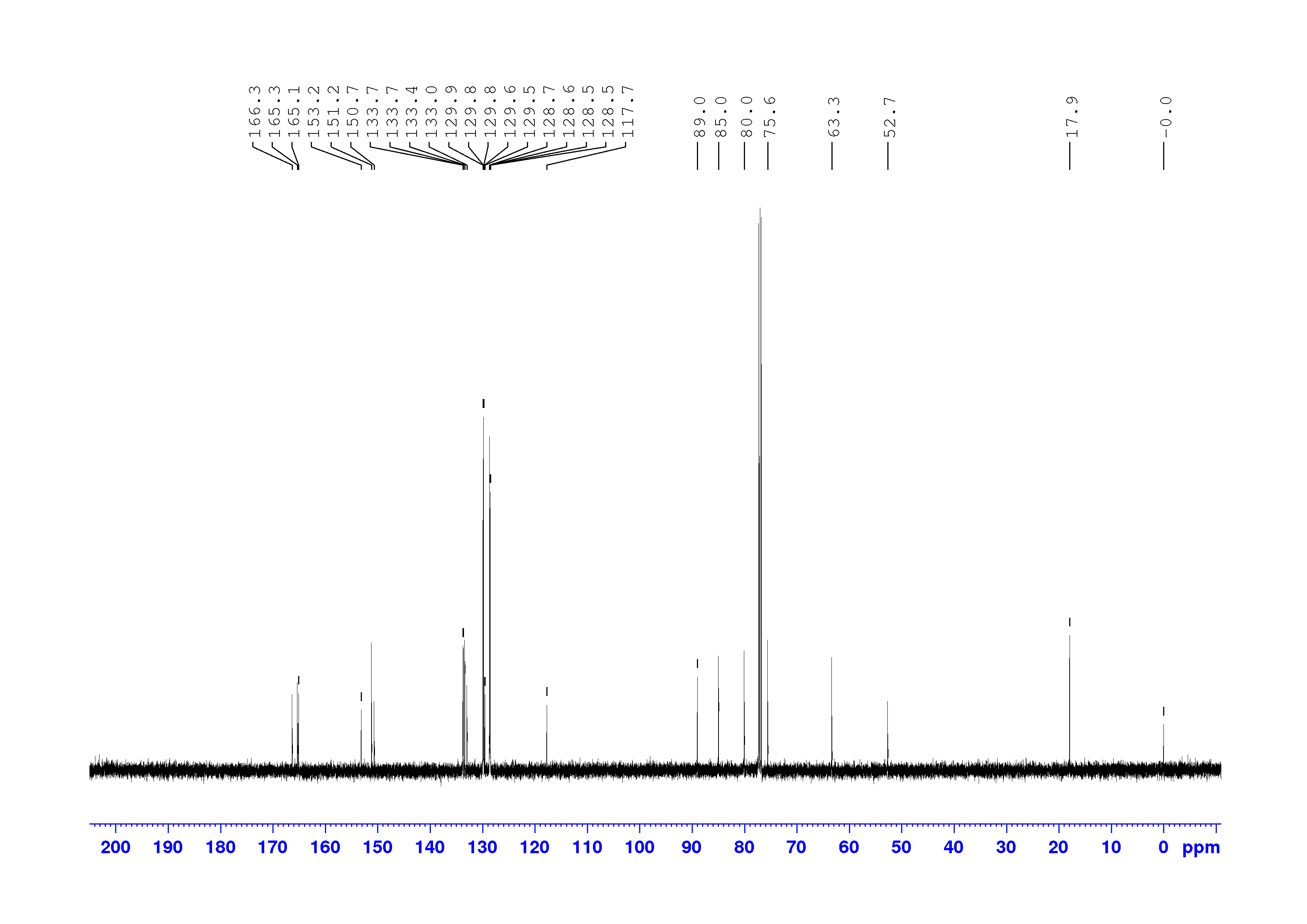
**

**(2*R*,3*R*,4*R*,5*R*)-5-((Benzoyloxy)methyl)-2-(4-chloro-5-iodo-7*H*-pyrrolo[2,3-*d*]pyrimidin-7-yl)-3-methyltetrahydrofuran-3,4-diyl dibenzoate (9) – 13C – CDCl_3_ – 125 MHz**

**
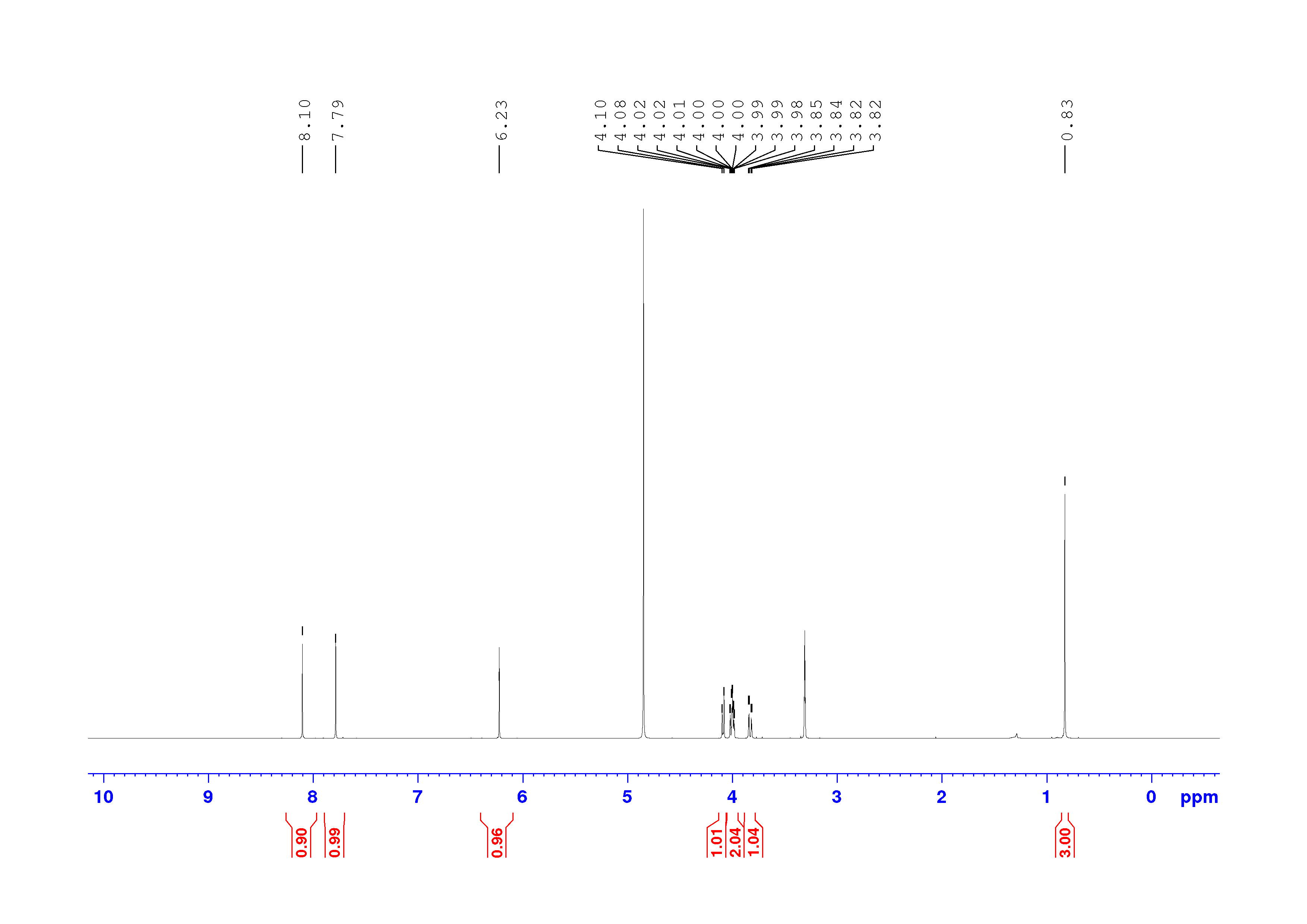
**

**(2*R*,3*R*,4*R*,5*R*)-2-(4-Amino-5-iodo-7*H*-pyrrolo[2,3-*d*]pyrimidin-7-yl)-5-(hydroxymethyl)-3-methyltetrahydrofuran-3,4-diol (13) – 1H – CD_3_OD – 500 MHz**

**
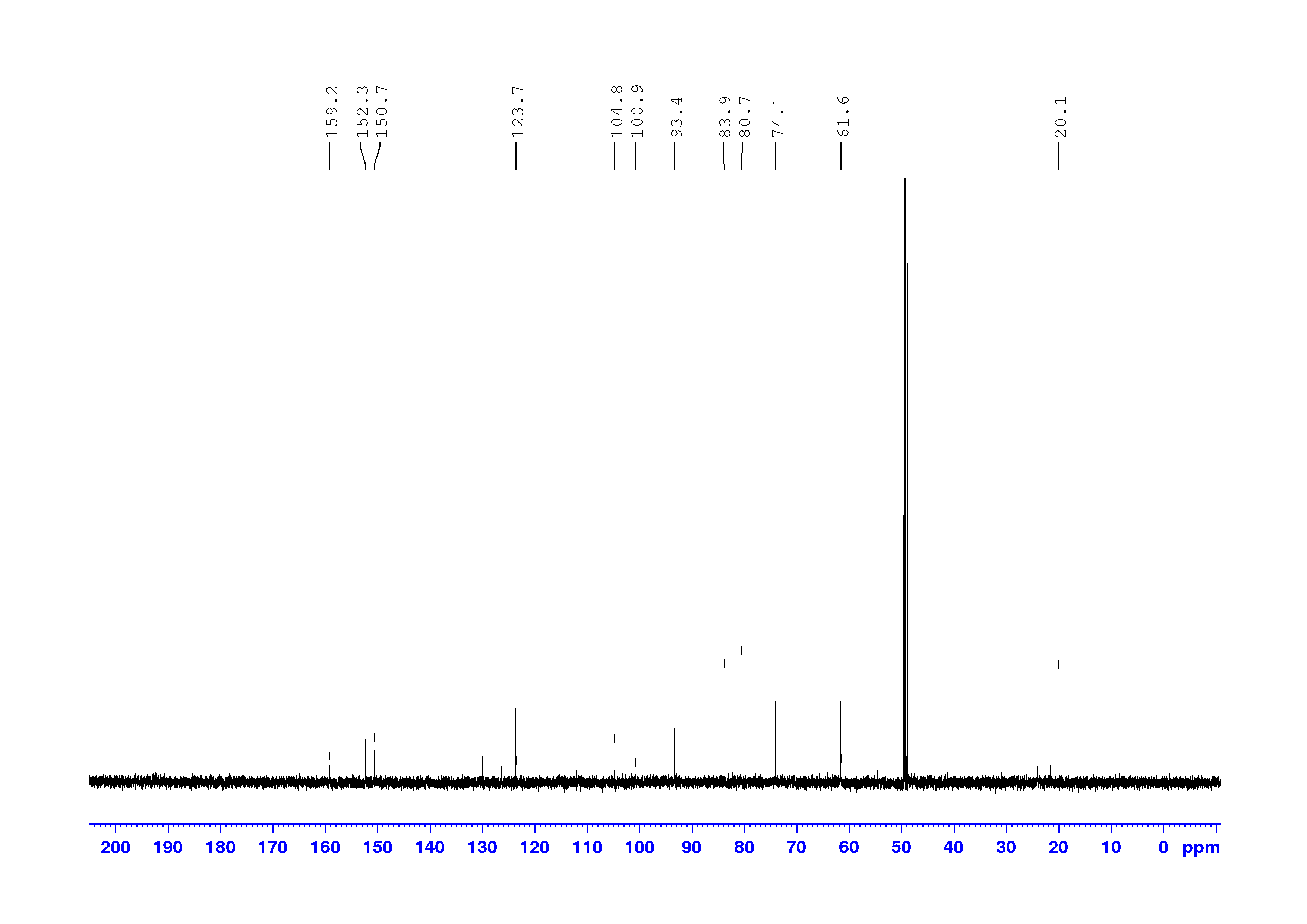
**

**(2*R*,3*R*,4*R*,5*R*)-2-(4-Amino-5-iodo-7*H*-pyrrolo[2,3-*d*]pyrimidin-7-yl)-5-(hydroxymethyl)-3-methyltetrahydrofuran-3,4-diol (13) – 13C – CD_3_OD – 125 MHz**

**
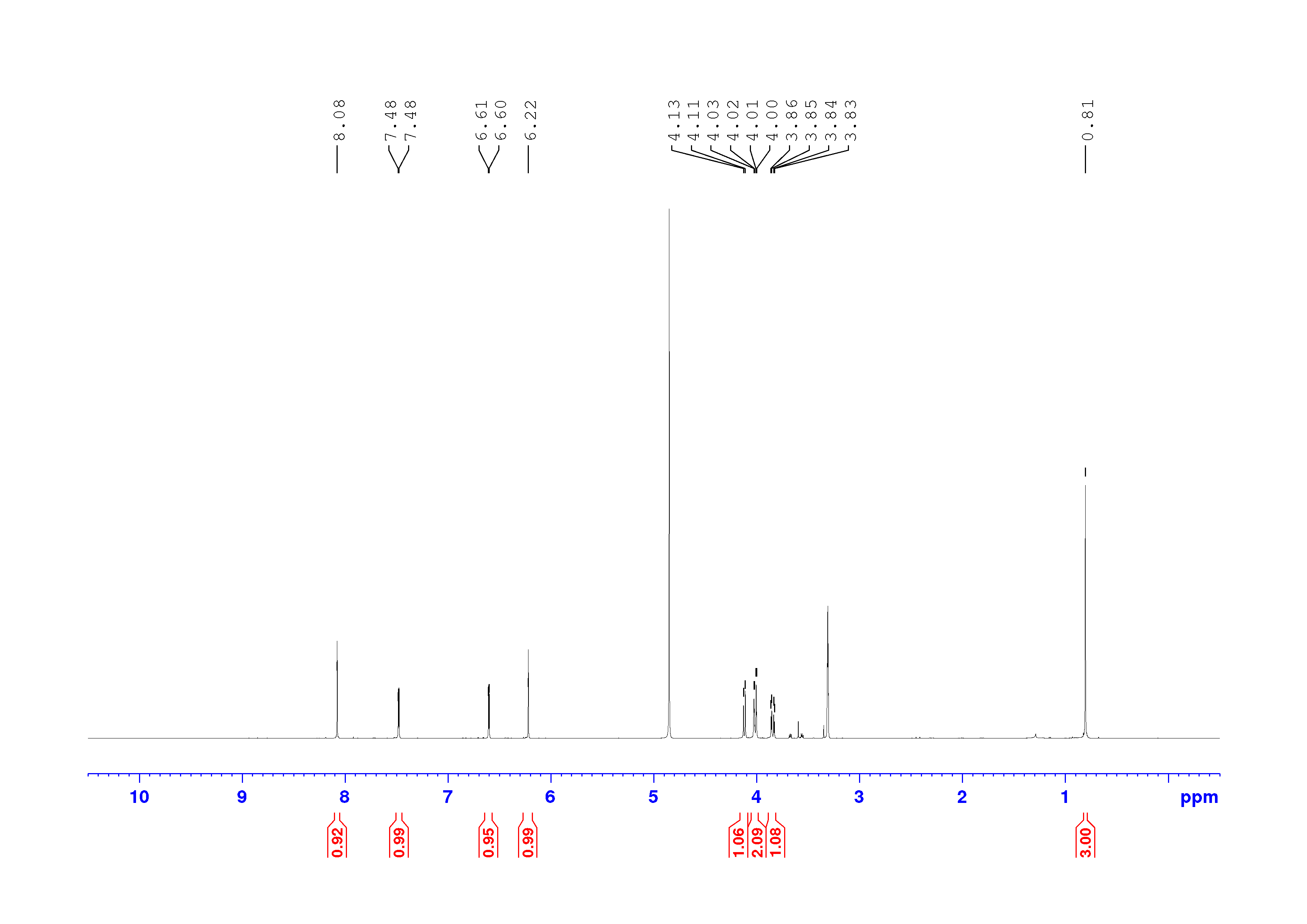
**

**7DMA (2) – 1H – CD_3_OD – 500 MHz**

**
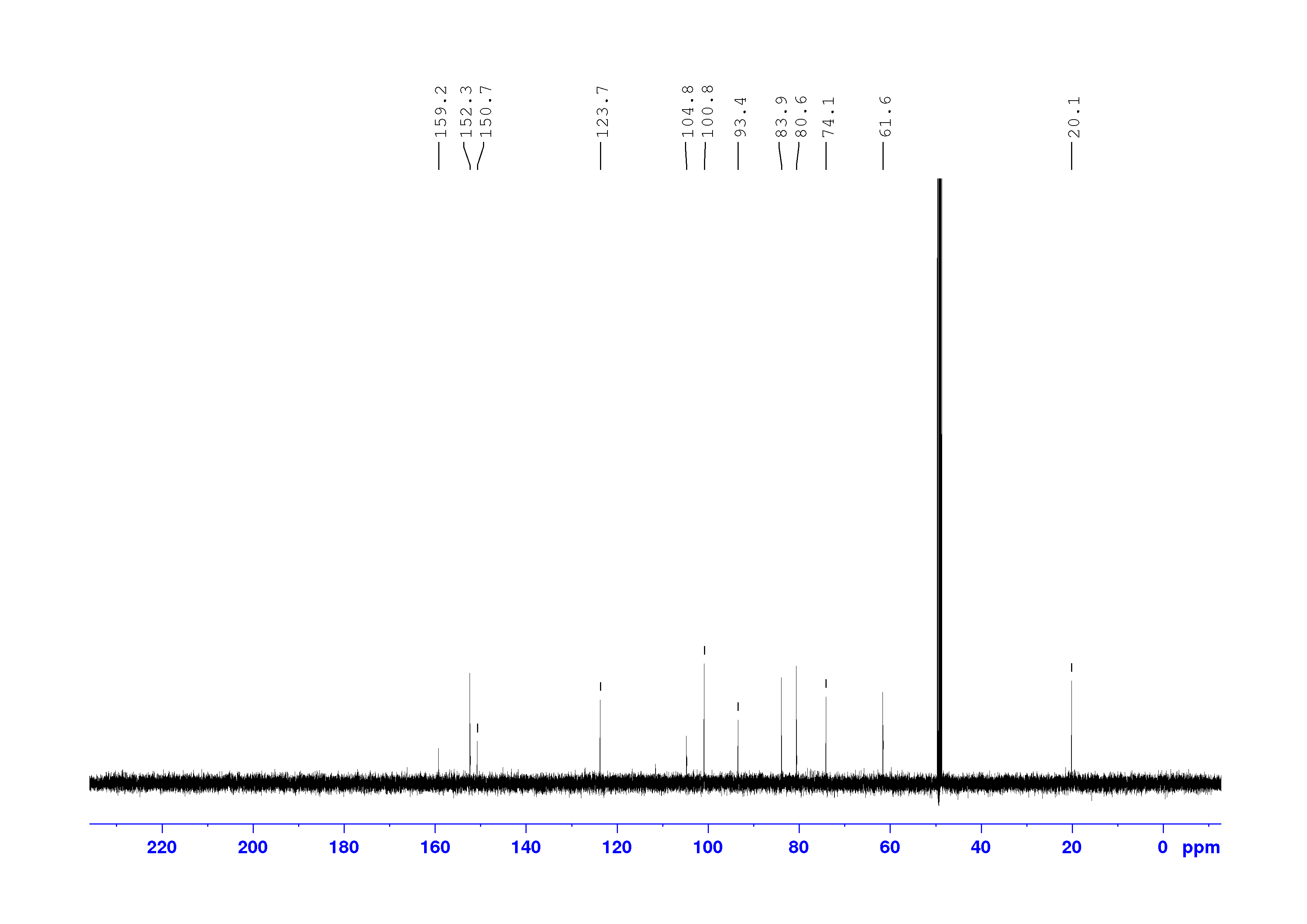
**

**7DMA (2) – 13C – CD_3_OD – 125 MHz**

**
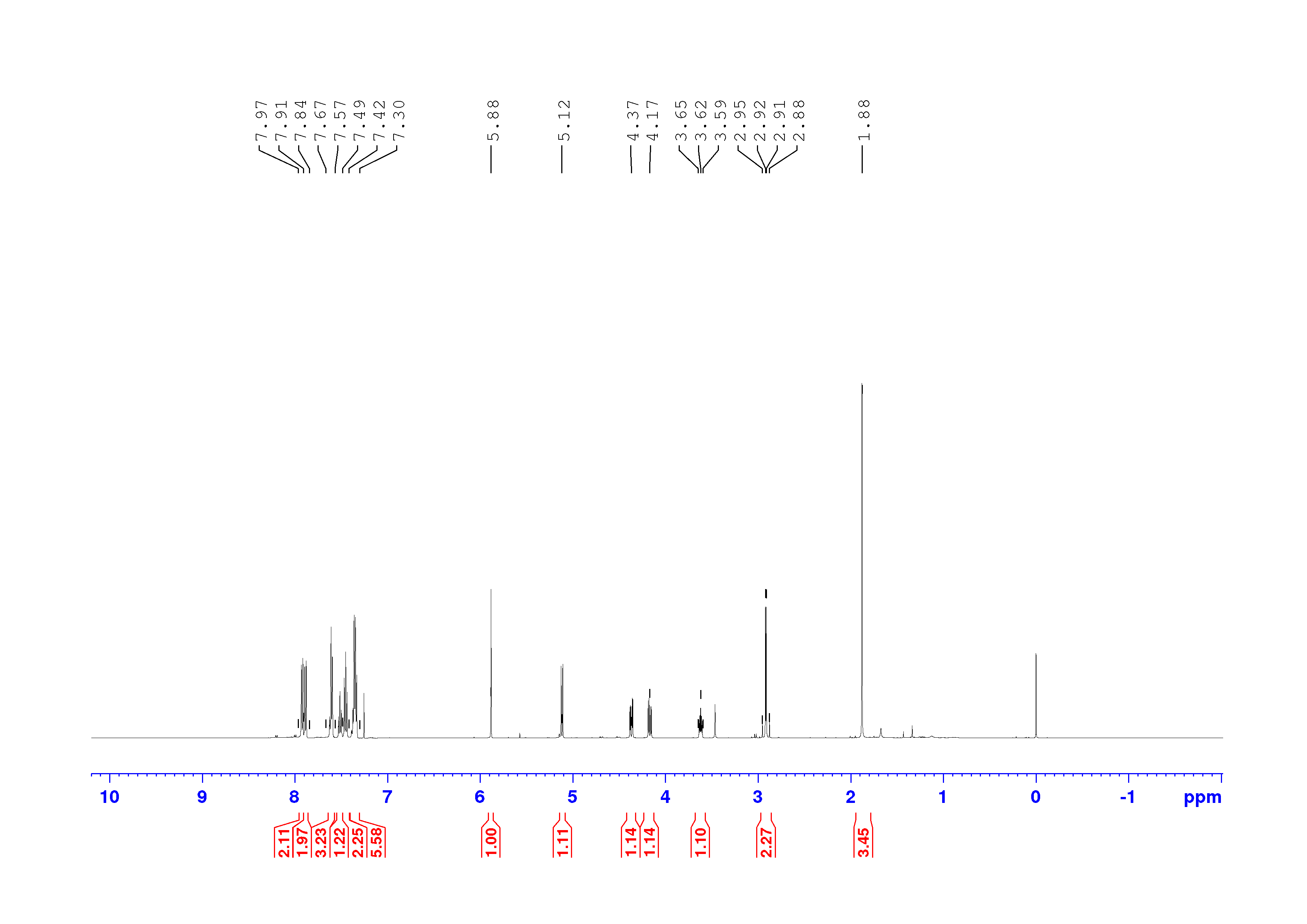
**

**(6-(Benzoyloxy)-2-(cyanomethyl)-6a-methyl-2-phenyltetrahydrofuro[2,3-*d*][1,3]dioxol-5-yl)methyl benzoate (10) – 1H – CDCl_3_ – 500 MHz**

**
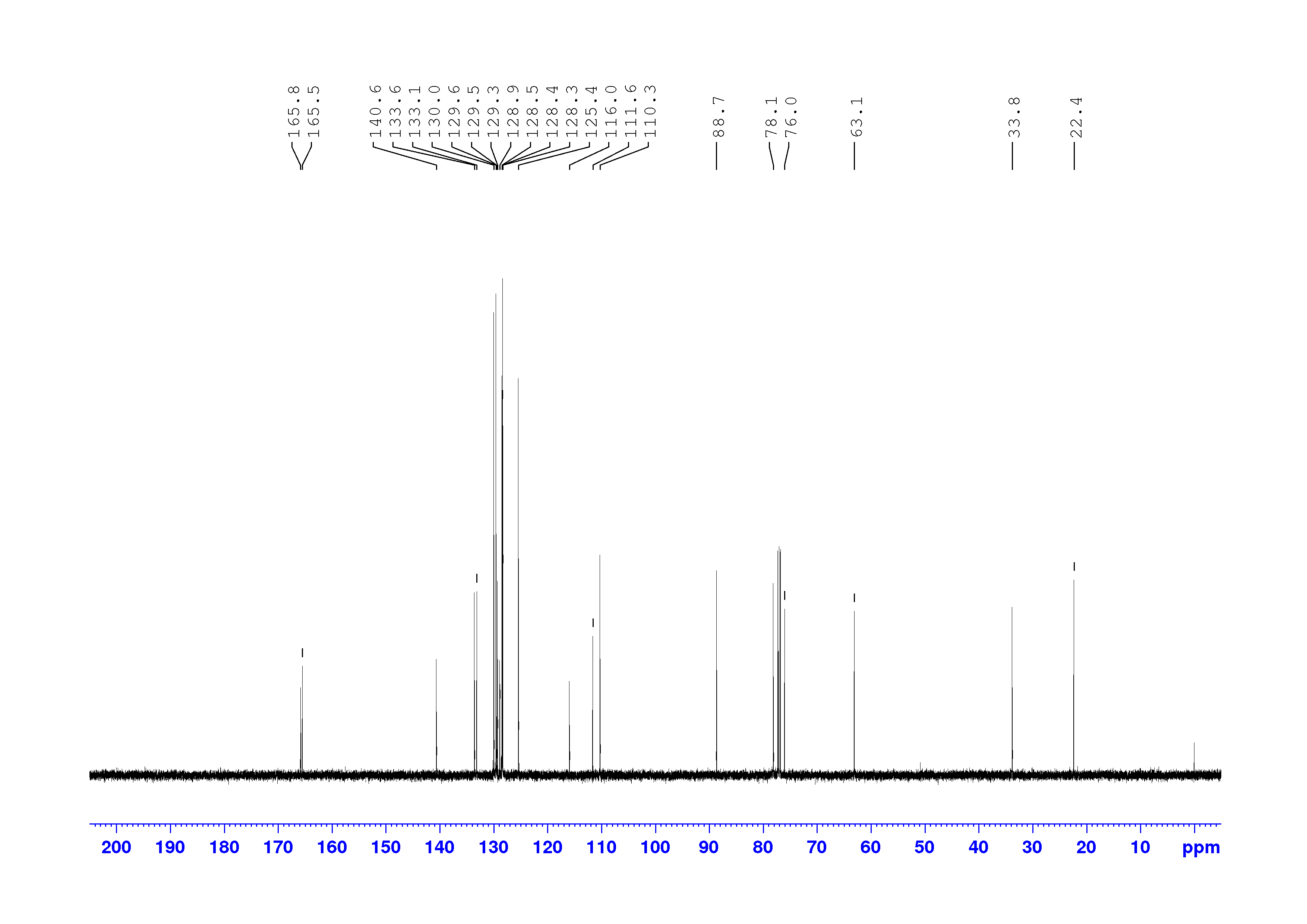
**

**(6-(Benzoyloxy)-2-(cyanomethyl)-6a-methyl-2-phenyltetrahydrofuro[2,3-*d*][1,3]dioxol-5-yl)methyl benzoate (10) – 13C – CDCl_3_ – 125 MHz**

**NOESY**

**NOESY**


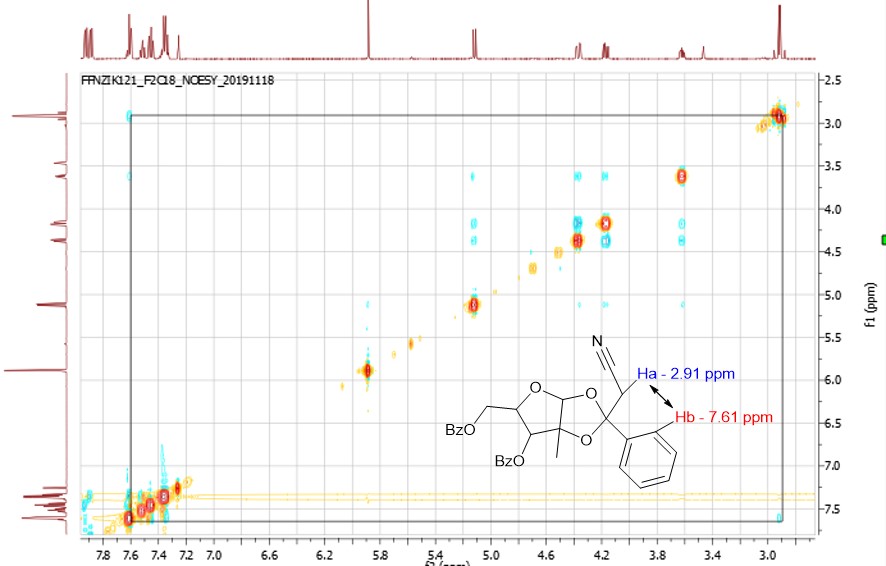


**HSQC**

CH_2_ – nitrile

CH_3_

**HSQC**

H5a’ X C5’

H5b’ X C5’

**HSQC**

H3’ X C3’

H4’ X C4’

**HSQC**

H1’ X C1’

**HMBC**

HMBC

CØ – ketal

CØ – nitrile

1. Hotchkiss, D. J., Soengas, R., Booth, K. v., Weymouth-Wilson, A. C., Eastwick-Field, V., and Fleet, G. W. J. (2007). Green aldose isomerisation: 2-C-methyl-1,4-lactones from the reaction of Amadori ketoses with calcium hydroxide. *Tetrahedron Lett* 48, 517–520. doi: 10.1016/j.tetlet.2006.11.137. [↑](#footnote-ref-1)
2. Storer, R., Moussa, A., Mathieu, S., and Qu, L. (2007). Process for the production of 3’-nucleoside prodrugs. [↑](#footnote-ref-2)
3. Pudlo, J. S., Nassiri, M. R., Kern, E. R., Wotring, L. L., Drach, J. C., and Townsend, L. B. (1990). Synthesis, Antiproliferative, and Antiviral Activity of Certain 4-Substituted and 4,5-Disubstituted 7-[(l,3-Dihydroxy-2-propoxy)methyl]pyrrolo[2,3-d jpyrimidines. doi: 10.1021/jm00169a028. [↑](#footnote-ref-3)
4. Nauš, P., Perlíková, P., Bourderioux, A., Pohl, R., Slavětínská, L., Votruba, I., et al. (2012). Sugar-modified derivatives of cytostatic 7-(het)aryl-7-deazaadenosines: 2′-C-methylribonucleosides, 2′-deoxy-2′-fluoroarabinonucleosides, arabinonucleosides and 2′-deoxyribonucleosides. *Bioorg Med Chem* 20, 5202–5214. doi: 10.1016/j.bmc.2012.07.003. [↑](#footnote-ref-4)
5. Eldrup, A. B., Prhavc, M., Brooks, J., Bhat, B., Prakash, T. P., Song, Q., et al. (2004). Structure−Activity Relationship of Heterobase-Modified 2‘- C -Methyl Ribonucleosides as Inhibitors of Hepatitis C Virus RNA Replication. *J Med Chem* 47, 5284–5297. doi: 10.1021/jm040068f. [↑](#footnote-ref-5)
